# Supplementary material for: Leucine-rich repeat kinase 2 impairs the release sites of Parkinson’s disease vulnerable dopamine axons
Source: Nat Commun. 2026 Jul 28;17:9165. doi: 10.1038/s41467-026-75194-3 (PMC13522591; doi:10.1038/s41467-026-75194-3)
Supplement: Supplementary file 1 [file 41467_2026_75194_MOESM1_ESM.pdf]

Supplementary Information for

Leucine-rich repeat kinase 2 impairs the release sites of Parkinson's disease vulnerable dopamine axons

Chuyu Chen *et al.*

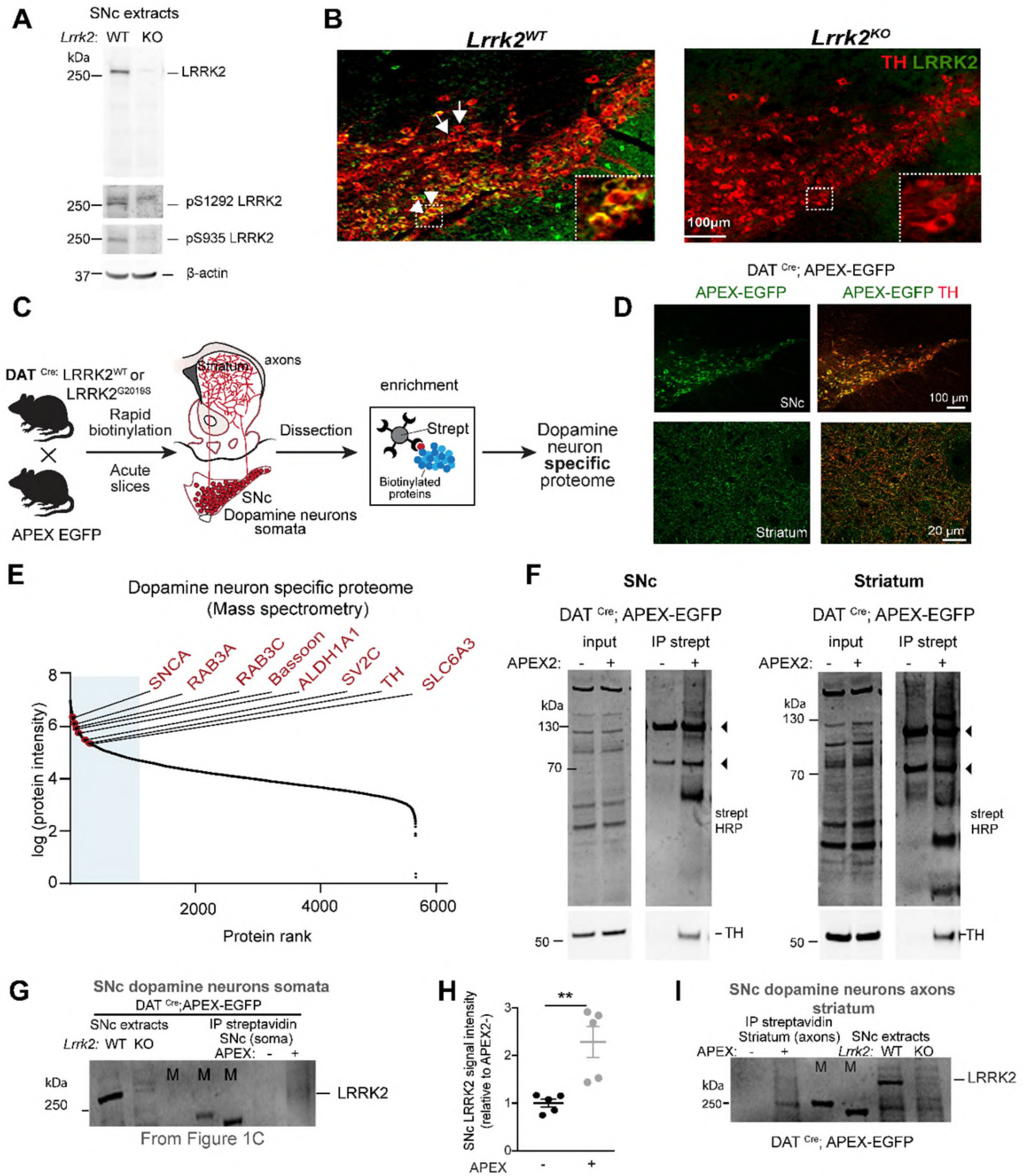

Supplementary Figure 1. LRRK2 protein is expressed in SNc dopamine neurons (linked to Figure 1).
A. WB analysis of SNc brain extracts from *Lrrk2^{WT}* and *Lrrk2^{KO}* mice probed with the antibodies indicated on the right. Note the absence of the LRRK2 signal in the *Lrrk2^{KO}* tissues. **B.** Brain sections from *Lrrk2^{WT}* and *Lrrk2^{KO}* mice were stained with the LRRK2 antibody from A, along with TH. Experiment was repeated independently 3 times with similar results **C.** Workflow of APEX2-based proximity labeling within genetically labeled dopamine neurons in the mouse brain. Acute brain slices from DAT^{Cre}; LRRK2^{WT} and DAT^{Cre}; LRRK2^{G2019S} mice crossed with the APEX2 EGFP reporter mouse line[46] were rapidly biotinylated, quenched, and dissected. Tissues were lysed and precipitated to remove free biotin. Tissue proteins were purified using streptavidin beads to enrich biotinylated proteins. On-bead digestion yielded peptides enriched from dopamine neurons, which were analyzed using mass spectrometry. **D.** Representative images showing the Cre-dependent expression of EGFP reporter along with TH immunostaining in the SNc and striatum of a

DAT^{Cre}; LRRK2^{WT}; APEX EGFP mouse. Experiment was repeated independently 3 times with similar results **E.** 85% of the top 55 mDA neuron marker genes (e.g., TH and DAT; SLC6A3) from a publicly available APEX2 proteome dataset (dataset identifier PXD026229 ProteomeXchange Consortium) were detected in all our mass spectrometry samples from DAT^{Cre}; APEX EGFP mice. 40% of these markers were among the top 20% (1105/5525) most highly expressed proteins by intensity (shaded light blue). **F.** WB analysis of biotinylated protein inputs and their streptavidin pulldowns from dissections of the indicated brain regions probed with antibodies as indicated on the right. Input: 10 μg of total protein (~1% of striatal lysate and ~5% of SNc); an equal volume of eluted proteins was loaded in each lane. Each lane represents pooled protein samples from 3 mice. **G**. Full gel from experiment presented in Figure 1C showing SNc extracts from *Lrrk2^{WT}* and *Lrrk2^{KO}* mice for reference of LRRK2 protein size. Each lane contains pooled samples from 3 mice. **H.** Quantification of LRRK2 intensity in DAT^{Cre} (APEX-) and DAT^{Cre}; APEX EGFP mice (APEX+) was normalized to the APEX2**-** condition. Each dot represents an independent experiment with 3mice pooled. Data represent mean±SEM. Bological replicates n=5 pooled samples/condition **= p0.0054, two-tailed unpaired t-test. **I.** WB showing streptavidin pull-downs from equal volumes of striatal proteins from DAT^{Cre} or DAT^{Cre}; APEX EGFP mice or DAT^{Cre} controls; SNc lysates from Lrrk2WT and Lrrk2KO are used as references for LRRK2 protein size. M, lanes loaded with molecular size markers. strept=streptavidin. Each lane contains pooled samples from 3 mice. Source data are provided as a Source Data file.

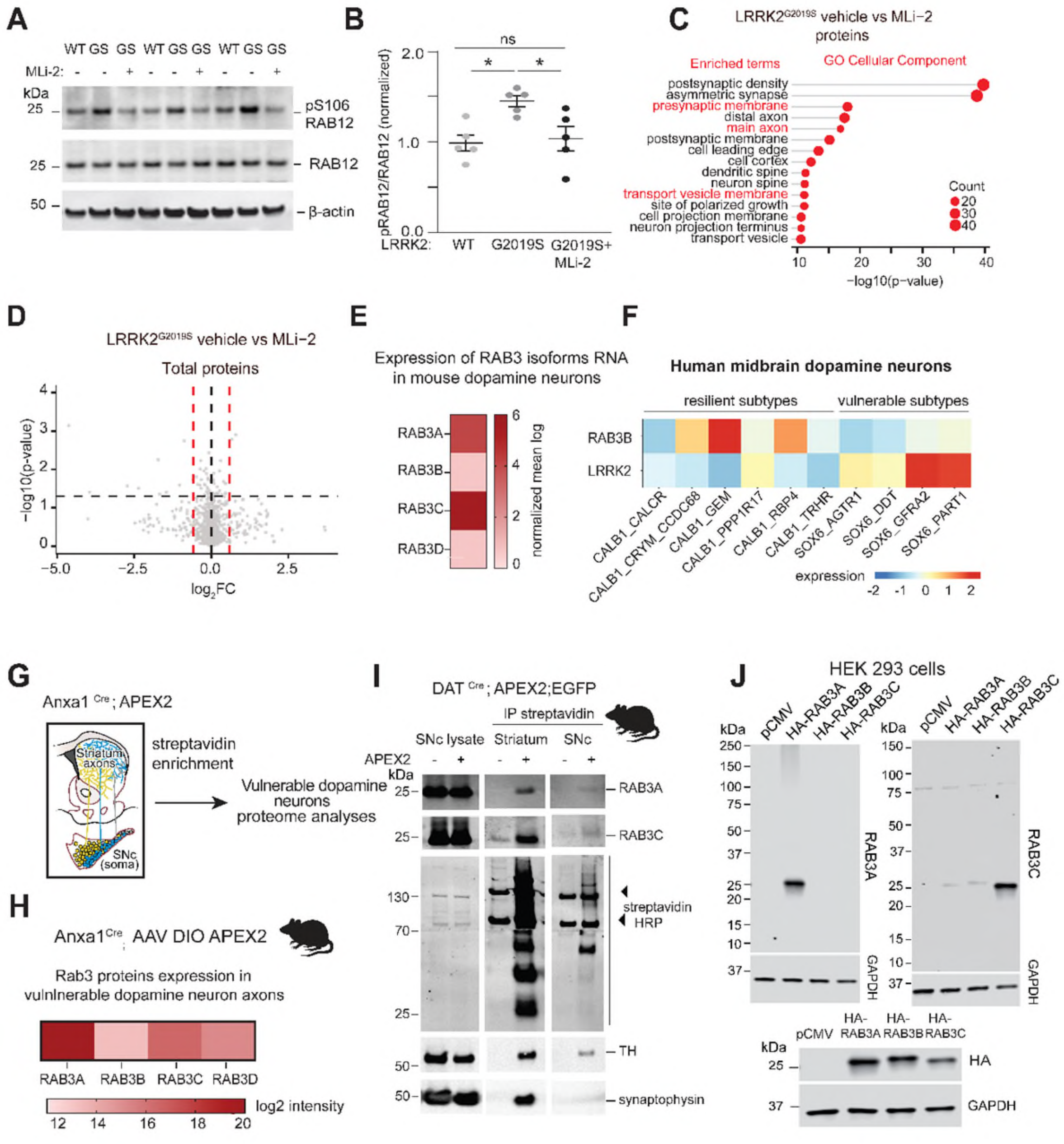

Supplementary Figure 2. Heterogeneous RAB3 isoform expression across dopamine neuron subtypes (linked to Figure 2).

A. WB analysis of SNc extracts from mice previously treated with MLi-2 (10 mg/kg) or vehicle for 2 h and probed for pS106 RAB12 (LRRK2 kinase target), total RAB12, and β-actin. **B.** Quantification of p-RAB12 band intensities normalized to total RAB12— Bological replicates n=5 mice/condition. Data are represented as mean±SEM (error bars). Asterisks denote statistical significance for Šídák's multiple-comparison tests after a two-way ANOVA. *p(WT vs GS)=0.0173, *p(GS vs GSMLi2)=0.0311. (Treatment factor $F(1,12)=8.614$ $p=0.0125$, Genotype factor $F(1, 12)=10.64$, $p=0.0068$) **C.** Gene Ontology analysis of proteins with at least one differentially regulated phosphopeptide in striatal synaptosomes from vehicle vs MLi2-treated $LRRK2^{G2019S}$ mice. The top 15 pathways significantly enriched in the cellular component term analysis (adjusted p-value ≤ 0.05 by hypergeometric test with HB correction). All enriched pathways have been uploaded to Zenodo. Link in the resources table.**D.** Volcano plot comparing the significantly altered total proteins ($|Log2FC| > 0.58$ and unadjusted p-value ≤ 0.05 by multiple two-tailed unpaired t-tests) between vehicle and MLi-2-treated $LRRK2^{G2019S}$ striatal synaptosomes. **E**. Heat map of the expression of RAB3 isoforms in a dataset of single-cell RNA sequencing.[9] The differential expression of RAB3 isoforms in TH+ SNc neurons compared to the remaining

non-TH SNc neurons is expressed as normalized mean log values (RAB3A = 4.11, RAB3B = 0.693, RAB3C = 5.59, and RAB3D = 0.693). **F.** Heat map illustrating the expression of LRRK2 and RAB3B isoforms in human dopamine neuron subpopulations from a single nucleus RNA sequencing dataset,[49] plotted with Shinny Cell.[99] **G.** Workflow of APEX2 experiment for dopamine neuron subpopulation-specific analysis with subcellular compartment resolution. Cre-dependent APEX2 expressing AAV (AAV5-CAG-DIO-APEX2-NES) was injected into the SNc of Anxa1^{Cre} LRRK2^{WT} mice for Anxa1+ dopamine neuron-specific APEX2 labeling. **H.** Relative expression of RAB3 protein isoforms in our Anxa1+ dopamine axon subcluster proteomic dataset (for further details see Supplementary Figure 6)**. I.** Equal amounts of eluted proteins from streptavidin pulldowns from the striatum and SNc from either a LRRK2^{WT} or a LRRK2^{G2019S} DAT^{Cre} mouse injected with APEX2 AAV. Each sample represents eluted proteins from 1 mouse. **J.** HEK293T cells were transiently transfected with either pCMV or HA-tagged RAB3 isoforms and blotted with isoform-specific antibodies or anti-HA, and with GAPDH for loading control. Equal expression of all isoforms is shown in the panel below. Source data are provided as a Source Data file.

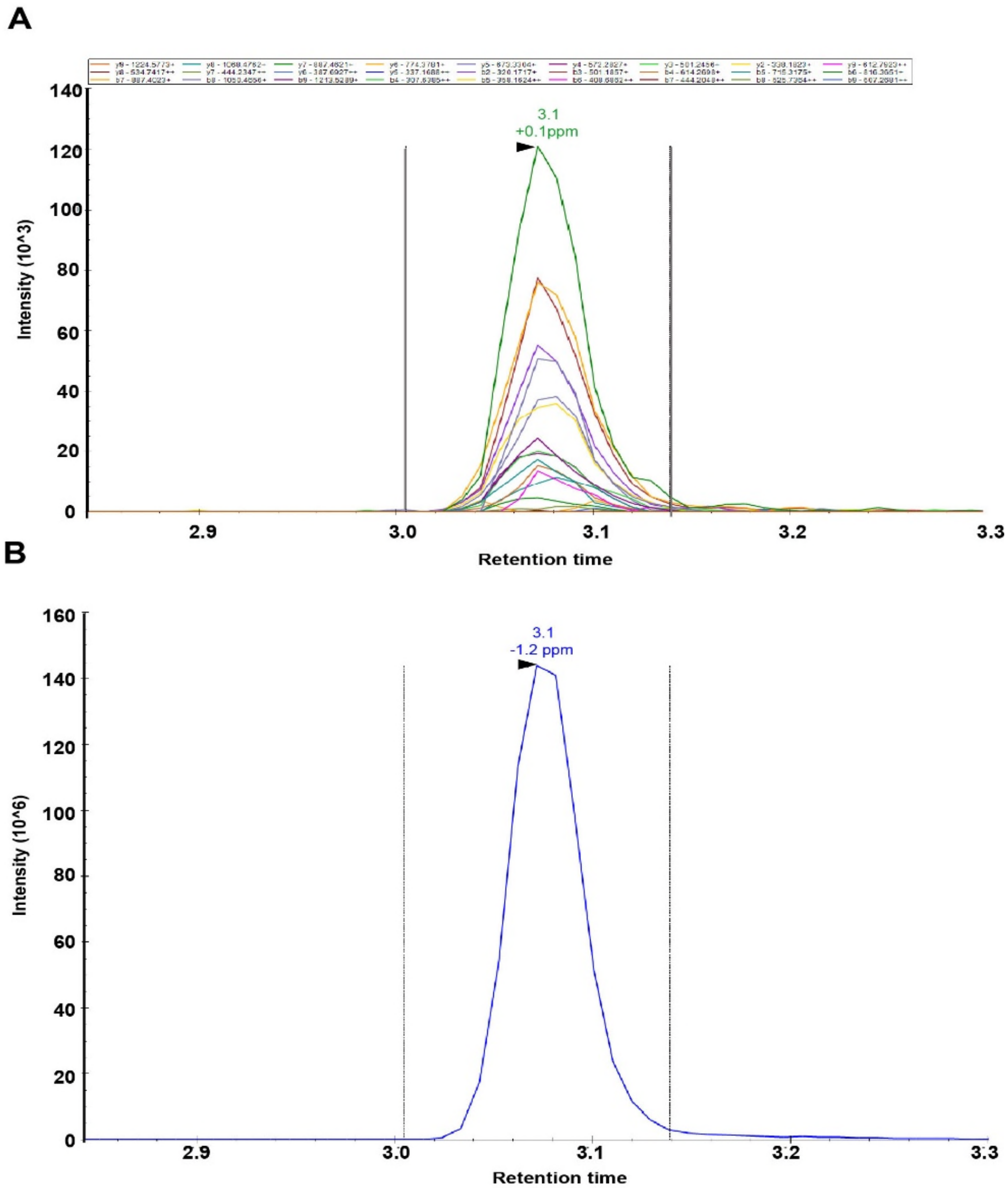

Supplementary Figure 3. Differential interactome of p-RAB3A and unphosphorylated RAB3A in extracts from mouse brain (linked to Figure 3).

A. Peptide fragment ions of the phosphopeptide; YRTITTAYR, of the 2+ precursor ion **(B).** which spans phosphorylation site T86 in recombinant p-Flag-RAB3A protein. All chromatograms elute at the same retention time, and peptide identifications are at a false-discovery rate of <1% (DIA-NN 1.9.2).

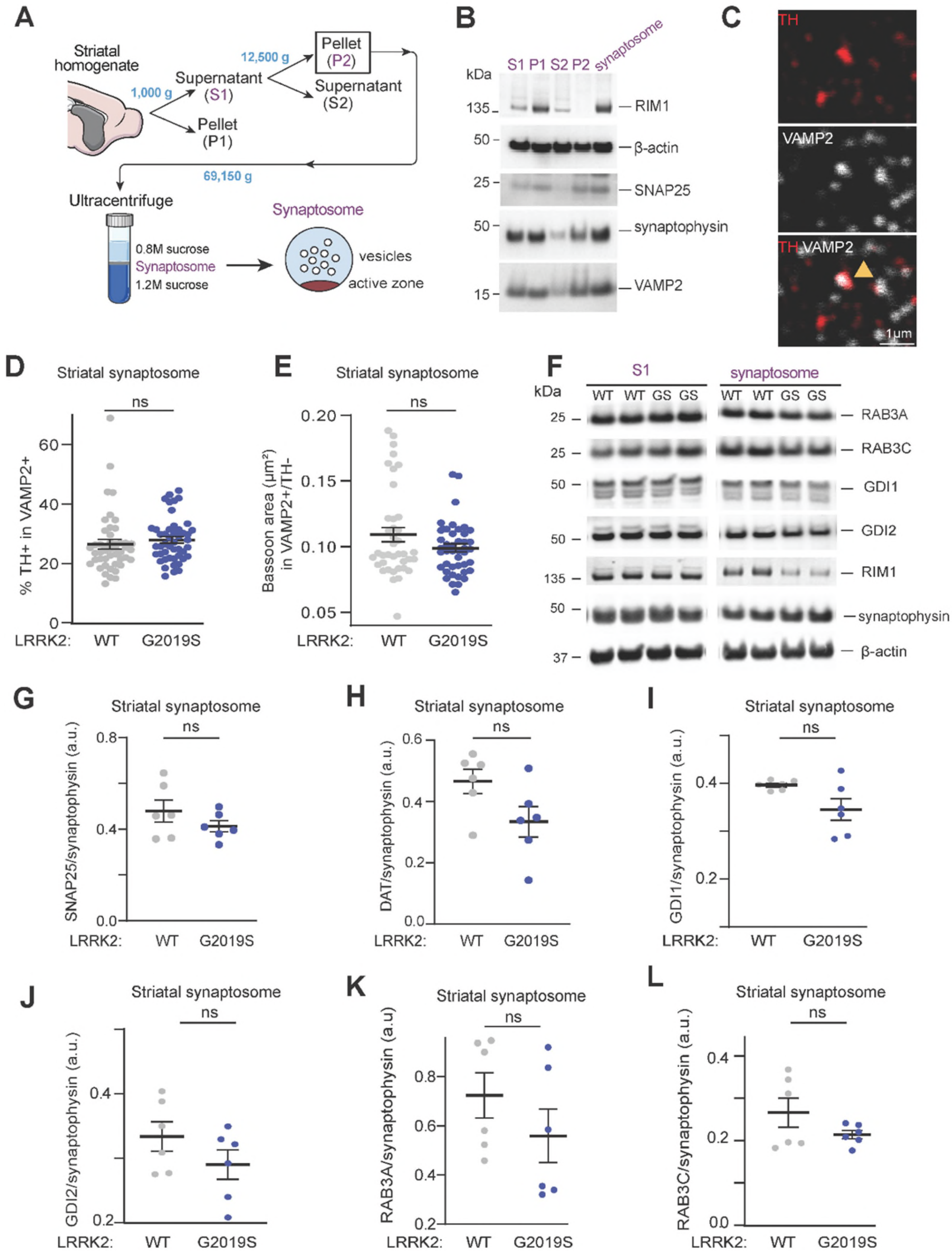

Supplementary Figure 4. LRRK2 alters the distribution and organization of active zones in striatal synaptosomes (linked to Figure 4).

A. Schematic representation of the steps for striatal synaptosome fraction preparation**. B.** WB analysis of selective fractions from striatal subcellular fractionation, probed with antibodies as indicated on the right. This analysis shows enrichment of

synaptic vesicles and active zone proteins. **C.** Confocal image of striatal synaptosomes stained with TH and VAMP2. A double positive synaptosome is indicated with the arrowhead. **D.** Quantification of the percentage of TH+/VAMP2+ striatal synaptosomes in LRRK2^{WT} and LRRK2^{G2019S} mice (denoted by WT and GS, respectively). Each circle represents the average result for an area containing approximately 1,000 synaptosomes. n=47 areas/4 mice per genotype. **E**. Quantification of bassoon area in VAMP2+ but TH- synaptosomes. Each circle represents the average of an image with ~1,000 synaptosomes, N_{WT}=39 areas/4 mice, N_{GS}=44 areas/4 mice. Data in D and E represent mean± SEM. Ns denotes no statistical significance after two-tailed unpaired t-tests. **F.** WB analysis of S1 and synaptosome fraction from LRRK2^{WT} and LRRK2^{G2019S} mice using the antibodies shown on the right. **G-L**. Quantification of the indicated protein signals in striatal synaptosomes from LRRK2^{WT} and LRRK2^{G2019S}. Data are mean± SEM. Ns =non-significant differences in comparisons after two-tailed unpaired t-tests. Bological replicates n= 6 mice/genotype. Equal amounts of proteins were loaded in all experiments, and duplicates represent biological replicates. Source data are provided as a Source Data file.

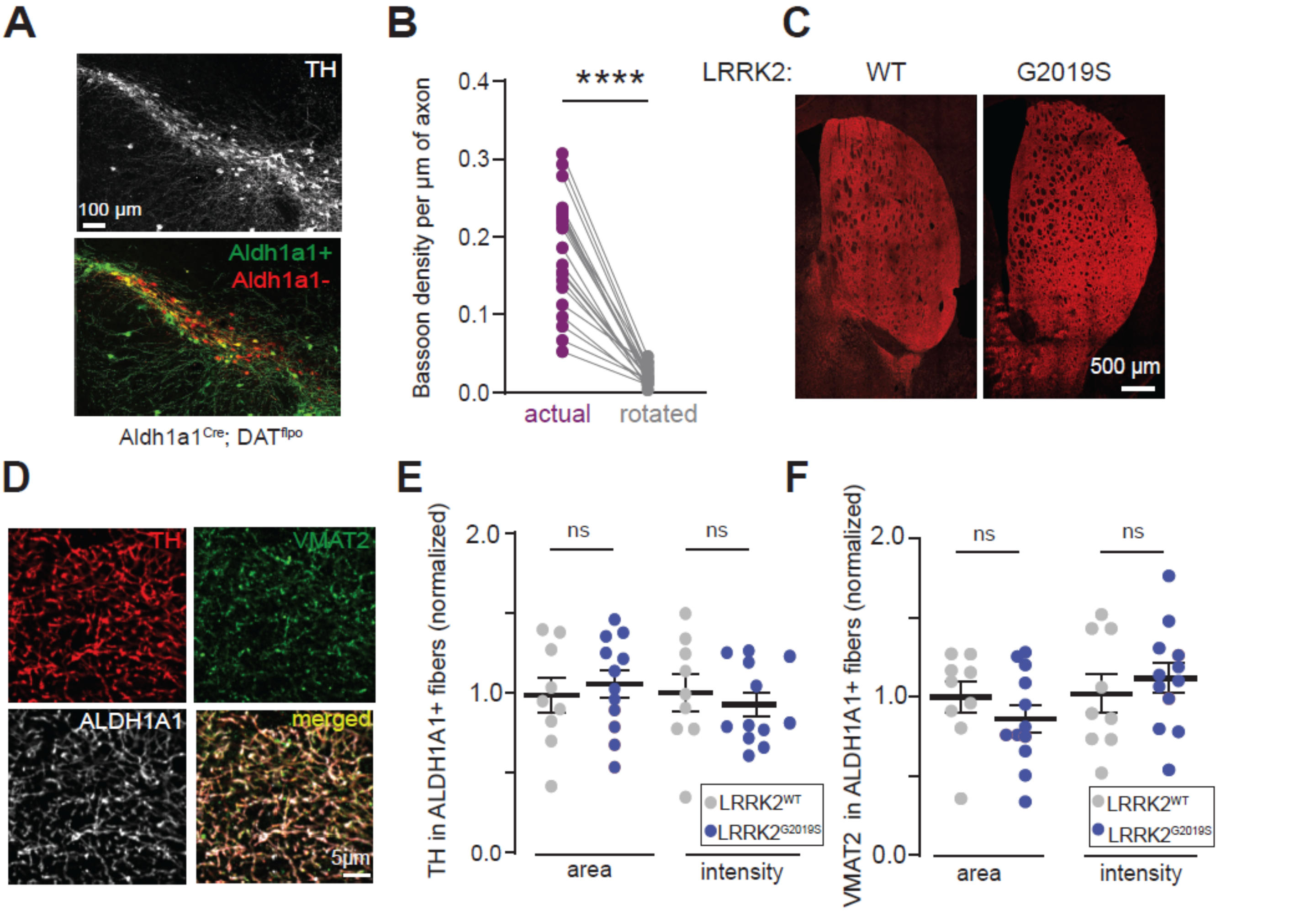

Supplementary Figure 5. LRRK2^{G2019S} changes the number and composition of active zones without affecting the overall integrity of TH fibers (linked to Figure 5).

A. SNc sections from Aldh1a1^{Cre}; DAT^{flpo} mice injected with viral plasmids stained with EGFP and mCherry to label Aldh1a1+ and Aldh1a1- dopamine neurons, respectively. Experiment was repeated independently 3 times with similar results **B**. Image with bassoon signal in control mice was rotated by 180°, whereas the eGFP channel remained unaltered. Quantification of bassoon density within dopamine axons before and after image rotation. Each circle represents the average result of a region containing 25,000-35,000 bassoon clusters. Data represent mean ±SEM. n=18 (3-6 sections/mouse, 4 mice). Significant differences in comparisons after two-tailed paired t-tests. **** p<0.0001. **C.** Representative low magnification striatal sections from 6-month-old LRRK2^{WT} and LRRK2^{G2019S} mice stained with TH. **D**. Representative high magnification dorsal striatal sections from adult (6 months) LRRK2^{WT} and LRRK2^{G2019S} mice stained with TH, ALDH1A1, and VMAT2 antibodies. **E**. Quantification of TH area and fluorescence intensity in ALDH1A1 immunostained dopamine axons across genotypes in the dorsal striatum. **F.** Average VMAT2 area and intensity in TH+/ALDH1A1+ dopamine axons across genotypes in dorsal striatum. For E and F, each dot represents the mean area or intensity of a striatal section, and data show mean±SEM; N_{WT} =9 sections/4 mice, N_{GS} =12 sections/ 5 mice. Ns: not significant with a two-tailed unpaired t-test**.** Source data are provided as a Source Data file.

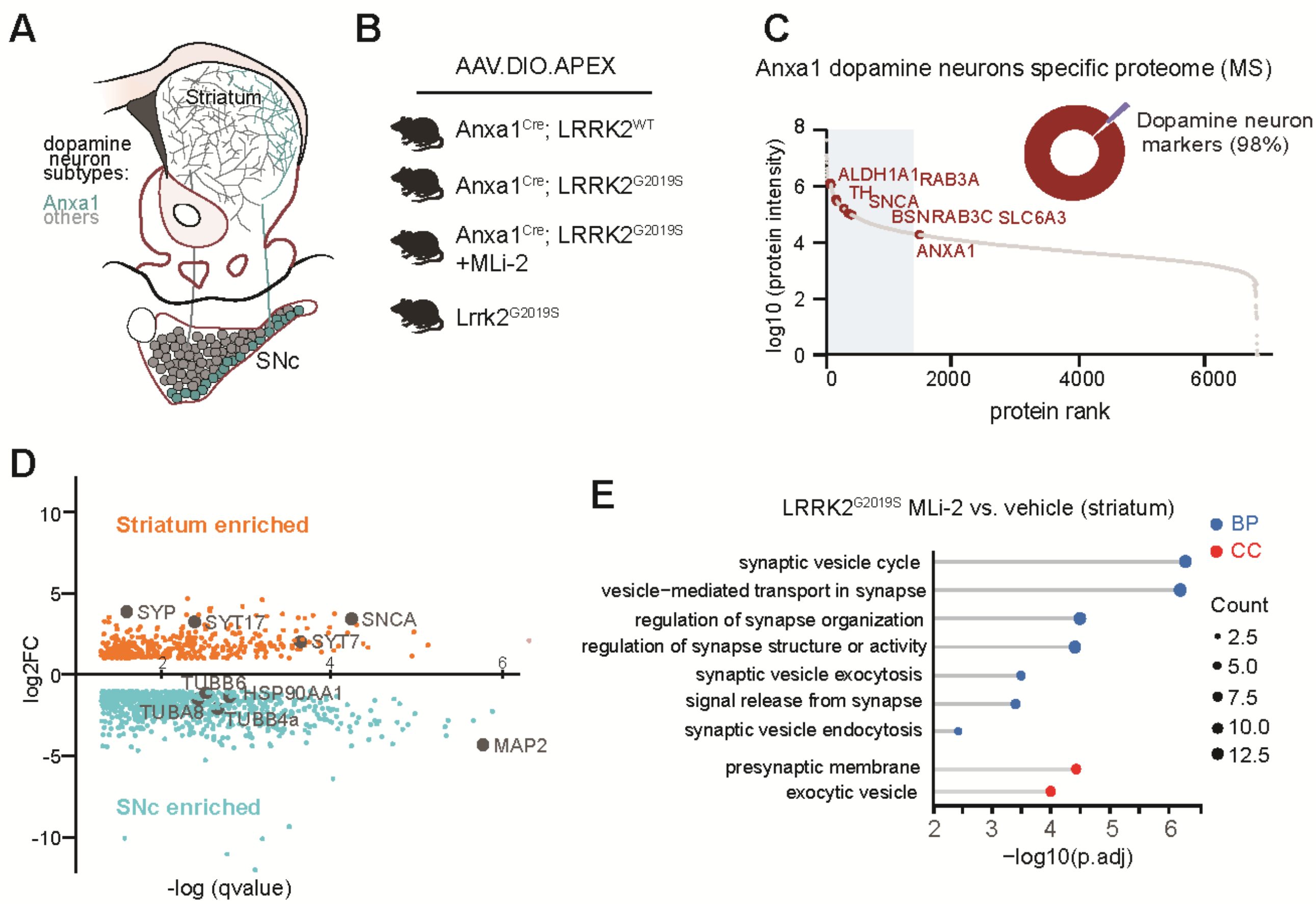

Supplementary Figure 6. Axonal and somatic proteomics of Anxa1+ neurons (linked to Figure 5).
A. Schematic illustrating the localization of Anxa1 dopamine neuron subtype in the mouse SNc and their projection patterns in the striatum. **B.** Experiment groups of APEX2-based proximity labeling within virally APEX2-labeled dopamine neurons in the mouse brain. **C**. Ranking plot showing the enrichment of dopamine neuron markers in the full dataset. 98% of the top 55 mDA neuron marker genes (e.g., TH and DAT (SLC6A3)) from a publicly available APEX2 proteome dataset (dataset identifier PXD026229 ProteomeXchange Consortium) were detected in all our mass spectrometry samples. Each sample is the eluted proteins from one mouse. To retain proteins specifically enriched by APEX2 labeling, we excluded proteins that were not significantly enriched in Anxa1^{Cre}; LRRK2^{G2019S} vehicle (APEX expressed) vs. control (no APEX expressed) q-value < 0.05, |log2FC| > 0) from both SNc and striatum datasets. In total 5567 proteins were identified after filtering **D.** Differential expression comparison of SNc and Striatum APEX+ streptavidin pulldown samples across the full dataset. Proteins colored orange or blue indicated significant enrichment in the Striatum vs. the SNc, respectively. (|Log2FC| > 0.58 and unadjusted p-value ≤ 0.05 by multiple two-tailed unpaired t-tests). Each sample represents eluted proteins from 1 mouse; striatum, n = 16; SNc, n = 17 **E.** Gene Ontology analysis of proteins differentially expressed in striatum from vehicle vs. MLi2-treated Anxa1iCre LRRK2^{G2019S} mice. Significantly altered proteins were defined using thresholds of |log2FC| > 0.58 and unadjusted p ≤ 0.05. Each sample consisted of eluted proteins from a single mouse. vehicle n=7, MLi2 n=5. BP (Biological Process) and CC (Cellular Component) refer to categories in GO. All enriched pathways have been uploaded to Zenodo and presented in Supplementary File 5; the link is available in the key resources table. Source data are provided as a Source Data file.

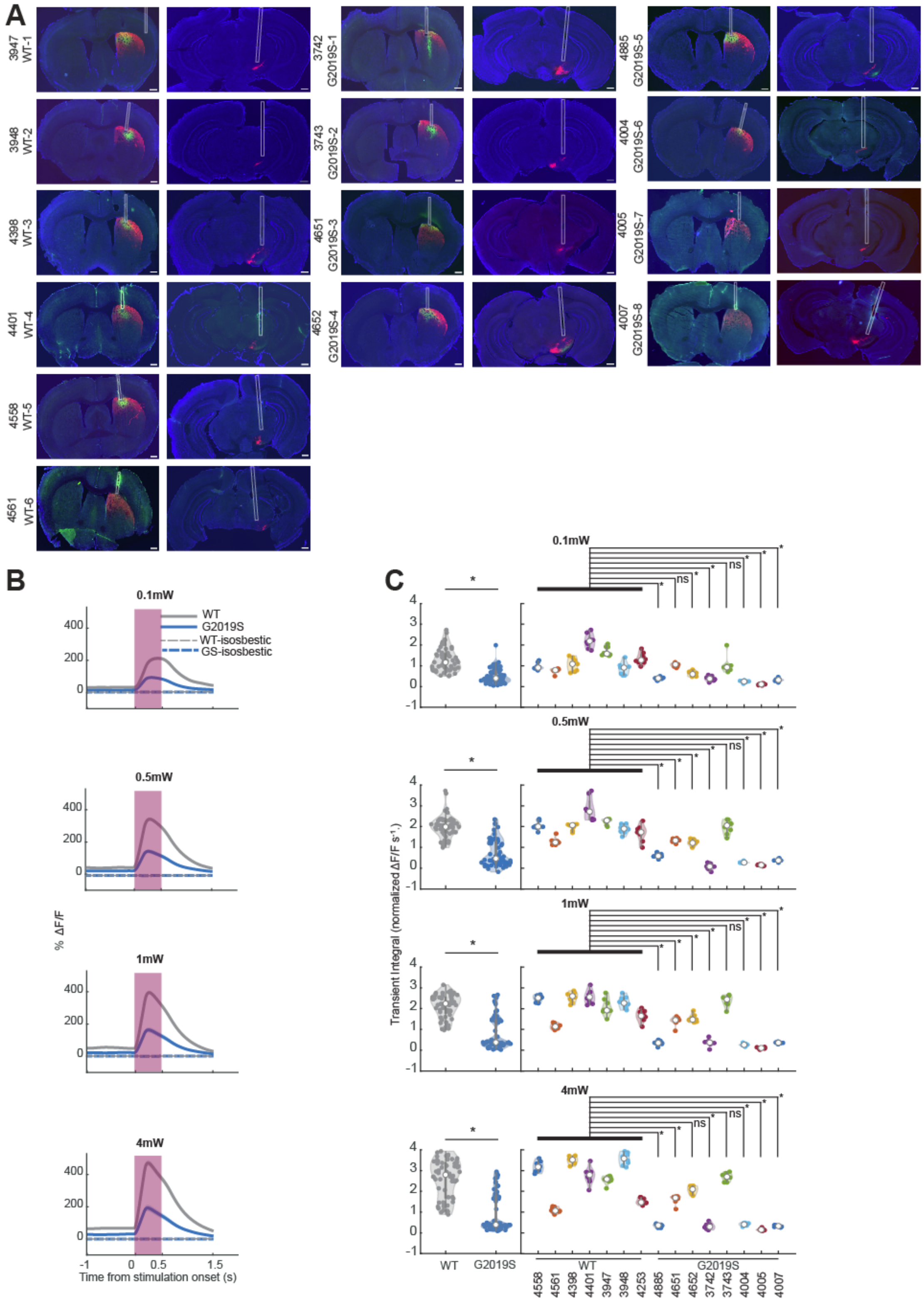

Supplementary Figure 7. Histology and power dependence for in vivo evoked dopamine measurements in the Anxa1+ subtype projection region of striatum (linked to Figure 6).
A. Histological verification of viral expression and fiber placement in each animal. Left two columns, coronal sections of the dorsal striatum region (dStr) and the Substantia Nigra pars compacta (SNc) of each LRRK2^{WT} mouse used in the analysis. Red fluorescence indicates the expression of ChRmine in the somas (SNc) and the axons (dStr) of Anxa1+ dopaminergic neurons. Green fluorescence indicates the expression of GRAB-DA3m in the dorsal striatum. White

rectangles indicate where the photometry (dStr) and optogenetics (SNc) fibers were placed. Right four columns, same as left, but in LRRK2^{G2019S} mice. Scale bar = 0.5 mm.

B. Same as Figure 6C but with 0.1mW, 0.5mW, 1mW, and 4mW optogenetic light powers from top row to bottom row (blue trace, LRRK2^{G2019S} mice = 8, n = 64 stimulations; grey trace, LRRK2^{WT} mice = 7, n = 56 stimulations). Shaded regions denote mean ± SEM across stimulations.

C. Same as Figure 6D but with 0.1mW, 0.5mW, 1mW, and 4mW optogenetic light powers from top row to bottom row. White dots denote group means and black bars denote 25th to 75th percentile. Left, group comparisons at each power (LRRK2^{G2019S} n = 64 stimulations per power, LRRK2^{WT} n = 56 stimulations per power; p-value (0.1 mW) = 1.29E-15, p-value (0.5 mW) = 1.10E-20, p-value (1 mW) = 1.22E-17, p-value (4 mW) = 2.39E-16, one-tailed, two-sampled t-test). Right, dopamine release response in individual mice. Numbers denote mouse ID. Asterisks (*) on the right denote p-value < 0.00625 from Welch's t-test with Bonferroni correction (α = 0.05/8 = 0.00625) comparing individual LRRK2^{G2019S} mouse to the LRRK2^{WT} group. Source data are provided as a Source Data file.

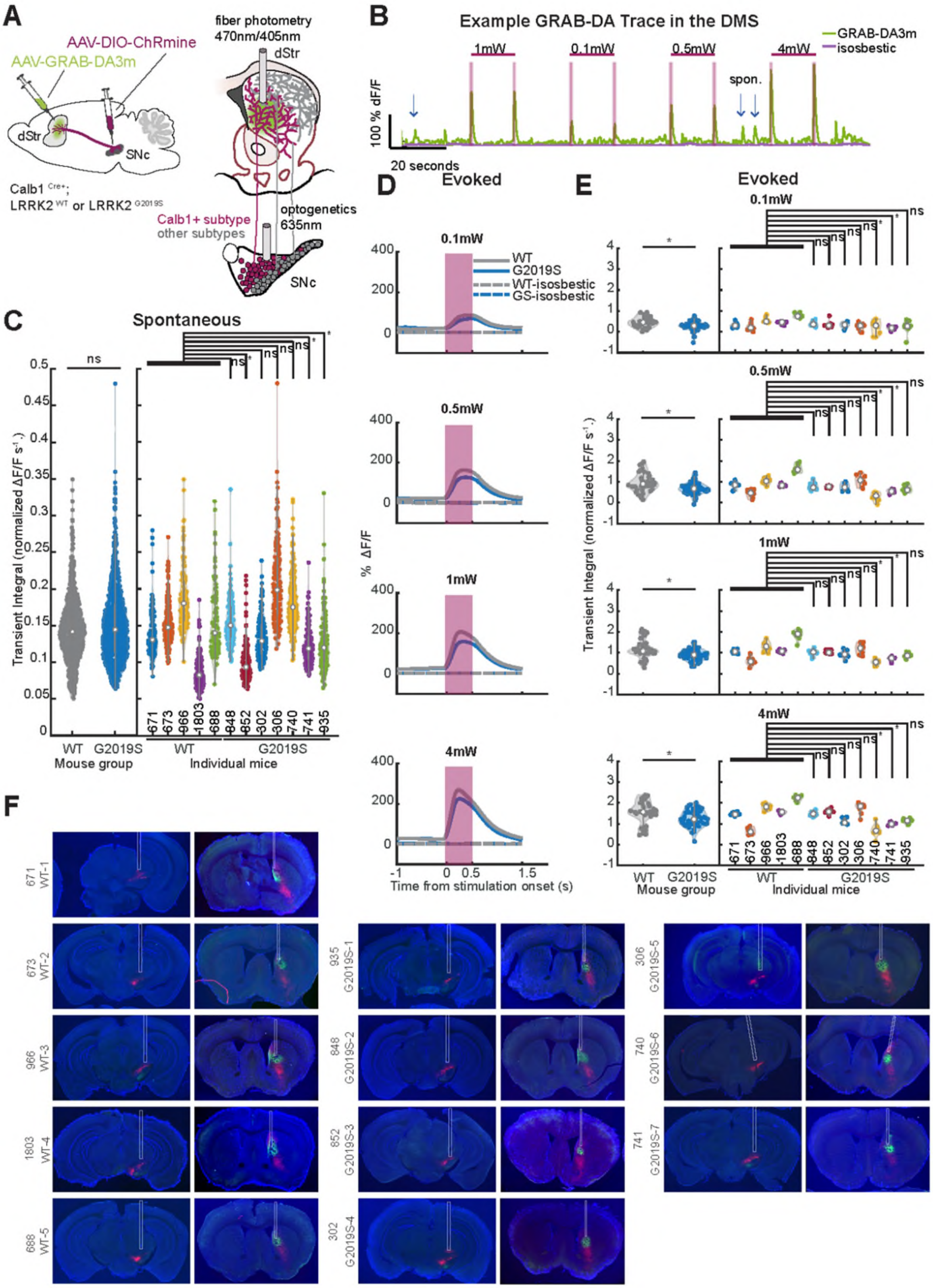

Supplementary Figure 8. In vivo evoked dopamine release is mildly reduced and spontaneous release is not reduced in the Calb1+ subtype projection region of striatum in LRRK2^{G2019S} compared to control mice.

A. Strategy used to label Calb+ dopamine neurons in the SNc of LRRK2^{WT} or LRRK2^{G2019S} mice with a red-shifted channelrhodopsin, ChRmine, and to express the fluorescent dopamine sensor, GRAB-DA3m, in the dorsal medial striatum

(DMS) for measuring dopamine release (ΔF/F). Left, sagittal view of the Calb1-specific ChRmine labeling in the SNc and their projection to the dorsal striatum; right, coronal view of overlapping ChRmine-expressing Calb1+ axonal projections and dopamine sensor expression the DMS.**B.** Example GRAB-DA3m recording from DMS during ChRmine activation of Calb1+ dopamine neurons (shaded in red). Blue arrow indicates example spontaneous dopamine transients.

C. Violin plots of integrated spontaneous dopamine transients in the DMS (normalized integral of GRAB-DA3m ΔF/F during significant dopamine transients selected by double-thresholding). Left, group-level comparisons ($LRRK2^{G2019S}$ n = 970 transients, WT n = 682 transients; p-value = 0.98, one-tailed, two-sampled t-test). Right, Violin plots of spontaneous (integrated) dopamine transients in individual mice. Asterisks (*) on the right denote p-value < 0.00714 from Welch's t-test with Bonferroni correction (α = 0.05/7 = 0.00714) comparing individual $LRRK2^{G2019S}$ mouse to the $LRRK2^{WT}$ group.

D. Evoked dopamine release in DMS triggered on optogenetic simulation: Average GRAB-DA3m ΔF/F triggered on optogenetic stimulation light onset with 0.1mW, 0.5mW, 1mW, and 4mW light power (blue trace, $LRRK2^{G2019S}$ mice = 7, n = 56 stimulations; grey trace, WT mice = 5, n = 40 stimulations; red shade, light stimulation time). Dashed lines, isosbestic control. Shaded regions denote mean ± SEM across stimulations

. **E.** Violin plots of optogenetically evoked (integrated) dopamine release response with 0.1mW, 0.5mW, 1mW, and 4mW stimulation light power (normalized integral of GRAB-DA3m ΔF/F during the optogenetic-stimulation light-on period). White dots denote group means and black bars denote 25th to 75th percentile. Left, group comparisons at each power ($LRRK2^{G2019S}$ n = 56 stimulations per power, $LRRK2^{WT}$ n = 40 stimulations per power; p-value (0.1mW) = 6.45E-05, p-value (0.5mW) = 0.0002, p-value (1mW) = 0.0001, p-value (4mW) = 0.001, one-tailed, two-sampled t-test). Right, dopamine release response in individual mice. Numbers denote mouse ID. Asterisks (*) on the right denote p-value < 0.00714 from Welch's t-test with Bonferroni correction (α = 0.05/7 = 0.00714) comparing individual $LRRK2^{G2019S}$ mouse to the $LRRK2^{WT}$ group. **F.** Histological verification of viral expression and fiber placement in each animal. Left two columns, coronal sections of the dorsal medial striatum region (DMS) and the Substantia Nigra pars compacta (SNc) of each $LRRK2^{WT}$ mouse used in the analysis. Red fluorescence indicates the expression of ChRmine in the somas (SNc) and the axons (DMS) of Calb1+ dopaminergic neurons. Green fluorescence indicates the expression of GRAB-DA3m in the dorsal medial striatum. White rectangles indicate where the photometry (DMS) and optogenetics (SNc) fibers were placed. Right four columns, same as left, but in $LRRK2^{G2019S}$ mice. Source data are provided as a Source Data file.

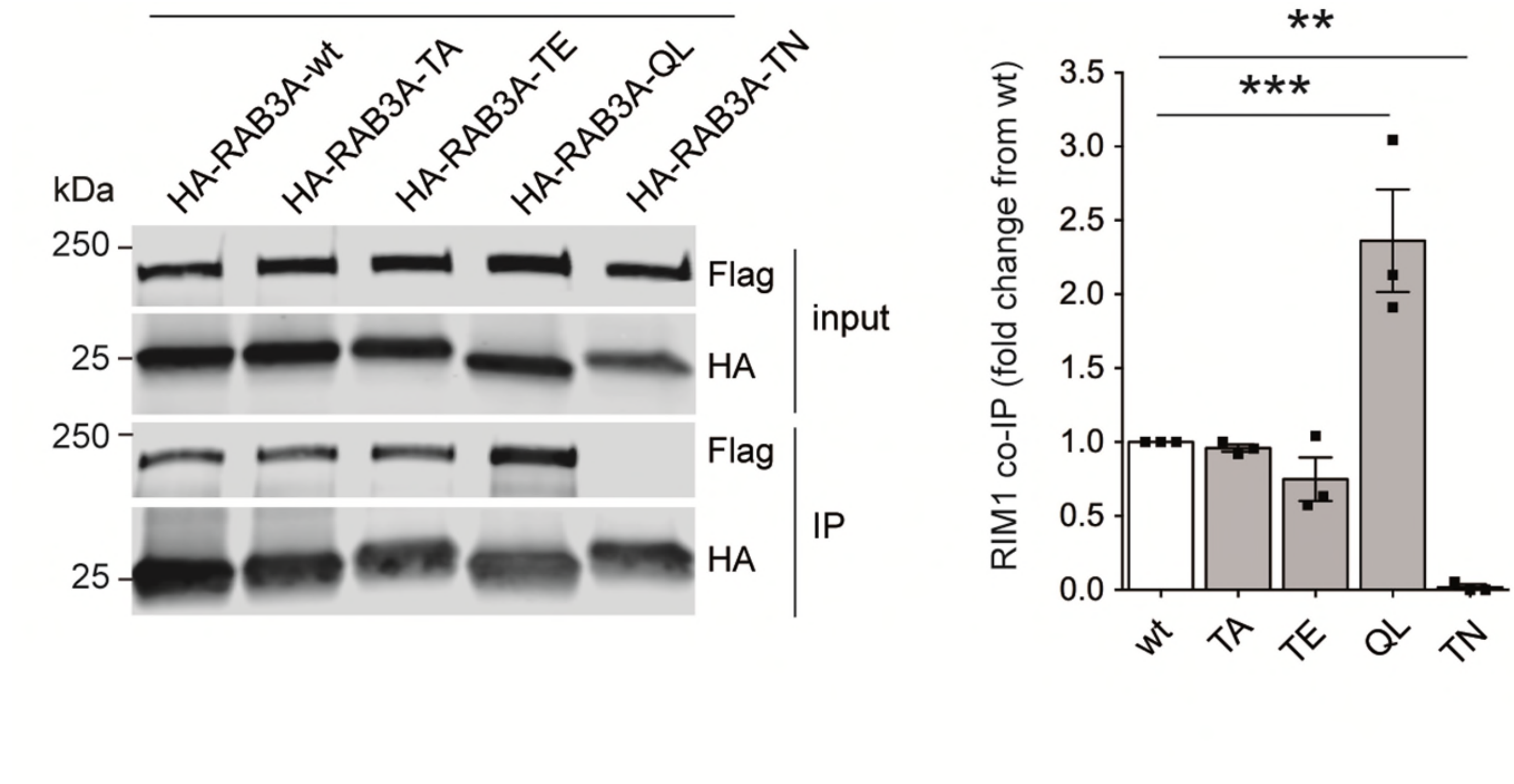

Supplementary Figure 9. Phosphomimetic RAB3 constructs fail to reproduce the effects of phosphorylated RAB3 proteins (linked to Figure 3).

A. HEK293 cells were transiently co-transfected with HA-tagged RAB3A constructs and myc-flag-RIM1. Extracts were subjected to pulldown with anti-HA beads, and input and immunoprecipitated material (IP) subjected to blotting with the indicated antibodies. WT, phospho-deficient (RAB3A-T86A) and phospho-mimic (RAB3A-T86E) RAB3A show similar binding to tagged RIM1. In contrast, and as expected, the RAB3A mutant mimicking the GTP-bound state (Q81L) shows increased RIM1 binding, whilst the RAB3A mutant mimicking the GDP-bound state (T36N) shows loss of RIM1 binding.
B. Quantification of experiments of the type shown in A. Co-immunoprecipitation of myc-flag-RIM1 with the indicated HA-tagged RAB3A constructs was normalized to that obtained with wild-type (wt) RAB3A. n=3 independent experiments (mean±SEM). Asterisk indicates statistical significance as determined by one-way ANOVA with Dunnett's multiple comparisons. ***p(WT vs QL)=0.0007; **p(WT vs TN)=0.007. Source data are provided as a Source Data file.

Supplementary Table 1

RESOURCE TYPE	***RESOURCE NAME***	***SOURCE***	***IDENTIFIER***
Antibody	Bassoon (SAP7F407)	Enzo, ADI-VAM-PS003	RRID:AB_10618753
	Tyrosine hydroxylase	SYSY, 213-104	RRID:AB_2619897
	Tyrosine hydroxylase	Millipore, MAB318	RRID:AB_2313764
	ALDH1A1	Abcam, ab52492	RRID:AB_867566
	ALDH1A1	Proteintech, 60171-1-Ig	RRID:AB_10693634
	anti-GFP	Invitrogen, A10262	RRID:AB_2534023
	anti-GFP	Abcam, ab6556	RRID:AB_305564
	anti-mCherry	Invitrogen, A11217	RRID:AB_2536611
	phospho-RAB12 (S106)	Abcam, ab256487	RRID:AB_2884880
	RAB12	Proteintech, 18843-1-AP	RRID:AB_10603469
	β-Actin	Sigma-Aldrich, A5441	RRID:AB_476744
	LRRK2/Dardarin cloneN241A/34	NeuroMab, 75-253	RRID:AB_10675136
	LRRK2/Dardarin cloneN137/6	NeuroMab, 75-188	RRID:AB_2234791
	LRRK2 (phospho S935)	Abcam, ab133450	RRID:AB_2732035
	LRRK2 (phospho S1292)	Abcam, ab203181	RRID:AB_2921223
	Streptavidin-HRP	Abcam, ab7403	
	RAB3A	Sigma-Aldrich, WH0005864M1	RRID:AB_1843230
	RAB3C	Proteintech, 10788-1-AP	RRID:AB_2177217
	Synaptophysin	Cell signaling, 4329	RRID:AB_1904154
	VAMP2	R&D systems, AF5136	RRID:AB_10571702
	SNAP25	Proteintech, 14903-1-AP	RRID:AB_2192051
	Dopamine Transporter	Sigma-Aldrich, MAB369	RRID:AB_2190413
	RIM1	Proteintech, 24576-1-AP	RRID:AB_2879618
	GDI1	Sigma-Aldrich, WH0002664M8	RRID:AB_1841744
	GDI2	Novus, NBP3-03650	RRID:AB_3532284
	VMAT2	Immunostar, 20042	RRID:AB_10013884
	GAPDH (6C5)	Santa Cruz, Sc-32233	RRID:AB_627679
	anti-Flag	Sigma, F1804	RRID:AB_262044
	anti-HA	Roche, 11867423001	RRID:AB_390918
	anti-c-myc	Sigma, M4439	RRID:AB_439694
	IRDye® 680RD Goat anti-rat IgG	LiCor, 926-68076	RRID:AB_10956590
	IRDye 800CW Goat anti-mouse IgG	LiCor, 926-32210	RRID:AB_3096013
	IRDye 800CW Goat anti-Rabbit IgG	LiCor, 926-32211	RRID:AB_621843
	IRDye® 680RD Goat anti- rabbit IgG	LiCor, 926-68071	RRID:AB_10956166
	Goat anti-Mouse IgG (H+L), HRP	Thermo Fischer Scientific, #626520	RRID:AB_2533947
	Goat anti-Rabbit IgG (H+L), HRP	Thermo Fischer Scientific, #656120	RRID:AB_2533967
	Goat anti-Chicken IgY (H+L), Alexa Fluor™ 488	Invitrogen, A11039	RRID:AB_2534096
	Donkey anti-Rabbit IgG (H+L) Highly Cross-Adsorbed, Alexa Fluor™ 488	Invitrogen, A21206	RRID:AB_2535792
	Goat anti-Guinea Pig IgG (H+L) Highly Cross-Adsorbed, Alexa Fluor™ 568	Invitrogen, A11075	RRID:AB_141954

	Donkey anti-Rabbit IgG (H+L) Highly Cross-Adsorbed, Alexa Fluor™ 647	Invitrogen, A31573	RRID:AB_2536183
	Donkey anti-Mouse IgG (H+L) Highly Cross-Adsorbed, Alexa Fluor™ 647	Invitrogen, A31571	RRID:AB_162542
	Cy™3 AffiniPure® Donkey Anti-Guinea Pig IgG (H+L)	Jackson Immuno Research, 706-165-148	RRID:AB_2340460
Virus strain	AAV8 hSyn-Con/Fon-YFP	Addgene #55650	RRID:Addgene_55650
	AAV8 Ef1a-Coff/Fon-mCherry	Addgene #137134	RRID:Addgene_137134
	AAV-CAG-DIO-APEX2NES	Addgene plasmid #79907	RRID:Addgene_79907
	pAAV-Ef1a-DIO-ChRmine-mScarlet-WPRE	Addgene #130998-AAV5	RRID:Addgene_130998
	pAAV-hSyn-GRAB-gDA3m	Addgene #208698-AAV1	RRID:Addgene_208698
Plasmids	pcDNA5D-frt/to-HA-RAB3A	mrcppureagents.dundee.ac.uk	DU36574
	pcDNA5D-frt/to-HA-RAB3A T86A	mrcppureagents.dundee.ac.uk	DU26352
	pcDNA5D-frt/to-HA-RAB3A T86E	mrcppureagents.dundee.ac.uk	DU26353
	pcDNA5D-frt/to-HA-RAB3A Q81L	mrcppureagents.dundee.ac.uk	DU36588
	pcDNA5D-frt/to-HA-RAB3A T36N	mrcppureagents.dundee.ac.uk	DU36587
	pCMV-HA-RAB3B	mrcppureagents.dundee.ac.uk	DU55007
	pCMV-HA-RAB3C	mrcppureagents.dundee.ac.uk	DU55048
	Myc-flag-RIMS1	OriGene	RC213013
	pET15b 6HIS MST3 TV2	mrcppureagents.dundee.ac.uk	DU62878
	FLAG-RAB3A	customized gene synthesis and generated by Genscript	
Chemical, peptide, or recombinant protein	MLi-2	Abcam, ab254528	
	Hydroxypropyl-β-Cyclodextrin	Cayman, 16169	
	Sodium chloride solution	Sigma-Aldrich, S5150	
	ProLong™ Diamond Antifade Mountant	Invitrogen, P36965	
	OCT embedding medium	Fisher Scientific #4585	
	Phosphate buffered saline	Sigma-Aldrich, P5493	
	Halt™ Protease and Phosphatase Inhibitor Cocktail, EDTA-free (100X)	Thermo Scientific, 78441	
	Tris Buffered Saline	Sigma-Aldrich, T5912	
	TWEEN® 20	Sigma-Aldrich, P1379	
	Sodium chloride	Sigma-Aldrich, S3014	
	Potassium chloride	Sigma-Aldrich, P9541	
	Sodium bicarbonate	Sigma-Aldrich S5761	
	Monosodium phosphate	Sigma-Aldrich S3139	
	Calcium chloride	Sigma-Aldrich C5670	
	D-(+)-Glucose	Sigma-Aldrich G7021	
	Sodium dodecyl sulfate solution	Sigma-Aldrich 71736-100ML	
	Ammonium bicarbonate	Fisher Chemical A643-500	
	MgCl2	Sigma-Aldrich M8266	
	HEPES	Sigma-Aldrich 54457	
	Antigen Unmasking Solution, Citrate-Based	Vector, H-3300	
	Triton X-100	Sigma-Aldrich, T8989	
	Glycerol	Sigma-Aldrich, G5516	
	Ethylenediaminetetraacetic acid disodium salt solution	Sigma-Aldrich, E7889	
	Methanol	Fisher Scientific #412	

Sucrose	Sigma-Aldrich, RDD023
Paraformaldehyde	Sigma-Aldrich, P6148
Choline chloride	Sigma-Aldrich, C7527
(+)-Sodium L-ascorbate	Sigma-Aldrich, A4034
Sodium azide	Sigma-Aldrich, S2002
Potassium phosphate monobasic	Sigma-Aldrich, 795488
Tris (1 M), pH 8.0	Invitrogen, AM9856
Trolox	Sigma-Aldrich, 238813
Sodium dodecyl sulfate solution	Sigma-Aldrich, 71736
Trichloroacetic acid	Sigma-Aldrich, T6399
urea	Thermo Scientific, 29700
Streptavidin magnetic beads	Thermo Scientific, 88817
Sodium phosphate, 0.5M buffer soln., pH 8.0	Thermo Scientific, J60825.AP
Bovine Serum Albumin	Sigma-Aldrich, A9647
Biotinyl tyramide	Sigma-Aldrich, SML2135
DL-Dithiothreitol	Sigma-Aldrich, D9779
2x YT Broth	FORMEDIUM, YDB0102
LB broth	Sigma-Aldrich, 102123542
IPTG	Fluorochem, M02726
Kanamycin Sulfate	Thermo Fisher Scientific, 11815032
Thrombin protease	Cytiva, 27-0846-01
Imidazole	Sigma-Aldrich, 102117349
Trizma base	Sigma-Aldrich, 0000349338
Magnesium chloride hexahydrate	Sigma-Aldrich, 102790662
2-Mercaptoethanol	Sigma-Aldrich, M3148
Adenosine 5'-triphosphate Disodium Salt Trihydrate	Thermo Fisher Scientific, BP413
Amicon® Ultra Centrifugal Filter, 10 kDa MWCO	Millipore, UFC9010
Phos-tag (TM) Acrylamide	FIJIFILM Wako, AAL-107
HisPur™ Ni-NTA Resin	Thermo Fisher Scientific, 88222
Pierce™ Anti-DYKDDDDK Magnetic Agarose	Thermo Scientific, A36798
Superdex 75 10/300 GL	Cytiva, 17517401
Mono Q 5/50 GL column	Cytiva, 17516601
cOmplete™, Mini, EDTA-free Protease Inhibitor Cocktail	Roche, 11836170001
PhosSTOP Phosphatase Inhibitor	Roche, 4906845001
12.5% SuperSep™ Phos-tag™	FUJIFLIM Wako, 199-18011
NuPAGE™ Bis-Tris Mini Protein Gels, 4–12%	Invitrogen™, NP0336BOX
iBlot™ 2 Transfer Stacks, nitrocellulose	Invitrogen™, IB23001
NuPAGE™ MES SDS Running Buffer (20X)	Invitrogen™, NP0002
NuPAGE™ Antioxidant	Invitrogen™, NP0005
NuPAGE™ Transfer Buffer (20X)	Invitrogen™, NP0006
NuPAGE™ LDS Sample Buffer (4X)	Invitrogen™, NP0008
NuPAGE™ Sample Reducing Agent (10X)	Invitrogen™, NP0009
10x TG transfer buffer	Fisher BioReagents, BP1306-4

	Pierce™ BCA Protein Assay Kits	Thermo Scientific™, 23225	
	Pierce™ Detergent Compatible Bradford Assay Kit	Thermo Scientific™, 23246	
	Restore™ Western Blot Stripping Buffer	Thermo Scientific™, 21059	
	BLUeye Prestained Protein Ladder	Sigma-Aldrich, 94964	
	Immobilon ECL Ultra Western HRP Substrate	Millipore, WBULS0500	
	Cell Lysis Buffer (10X)	Cell Signaling, 9803	
	OCT embedding medium	Fisher Scientific, 4585	
	Superfrost™ Clipped Corner Slides	Fisher Scientific, 12-550-016	
	Nitrocellulose Membranes, 0.45 µm	Fisher Scientific, 88018	
	low glucose DMEM	Gibco, 11885-084	
	high glucose DMEM	Gibco, 11965092	
	Fetal bovine serum	Gibco, A5256801	
	MEM non-essential amino acids	Gibco, 11140050	
	trypsin/EDTA	Gibco, 25200072	
	One Shot TOP10 chemically competent E. coli	Invitrogen, C404003	
	ZymoPURE II Plasmid MidiPrep kits	Zymo Research, D4201	
	LipoD293	Signagen Laboratories, SL100668	
	Complete, Mini, EDTA-free protease inhibitor cocktail	Roche,11836170001	
	Magnetic anti-HA beads	Pierce, 88836	
	Acetonitrile ≥99.9%	VWR, 1.00030.2500	
	Formic Acid, 99.0+%, Optima™ LC/MS Grade	Thermo Fisher Scientific, A117-50	
	Water, for HPLC-MS	Fisher Scientific, 10777404	
	EPA Screw Vial Assembled Kit, 20mL amber glass EPA	Thermo Scientific, 11543750	
	Sodium Dodecyl Sulfate (SDS)Micropellets	Thermo Fisher Scientific, 15450685	
	Hepes 99.6%	Formedium, HEPES10	
	Bond-Breaker™ TCEP Solution, Neutral pH	Thermo Scientific, 77720	
	Iodoacetamide, BioUltra	Sigma-Aldrich, I1149-25G	
	Trifluoroacetic acid (TFA)	Sigma-Aldrich, 302031-100ML	
	Triethylammonium bicarbonate buffer (TEABC)	Sigma-Aldrich, T7408-500ML	
	Trypsin/Lys-C Mix	Thermo Fisher Scientific, #15956915	
	n-Dodecyl-beta-Maltoside Detergent (DDM)	Thermo Scientific, 89902	
	S-Trap micro columns	Protifi, C02-micro-80	
	Complete EDTA-free protease inhibitor cocktail	Roche, 11873580001	
	PhosSTOP phosphatase inhibitor cocktail tablets	Roche, 4906837001	
	Micro Tube 2ml Low Binding	Sarstedt, 72.695.600	
	Eppendorf twin.tec PCR Plates LoBind (96-well)	Thermo Fisher Scientific, 15280735.	
	Mounting Medium with DAPI	VECTASHIELD, H-2000	
	C&B-Metabond Dental Cement	Fisher Scientific NC0877382	
Mouse model	*LRRK2 G2019S*	Jackson Laboratory 030961	RRID:IMSR_JAX:030961
	C57BL/6	Jackson Laboratory 000664	RRID:IMSR_JAX:000664

	LRRK2 KO	Jackson Laboratory 016121	RRID:IMSR_JAX:016121
	Aldh1a1-cre	Transgenic and Targeted Mutagenesis Laboratory, Northwestern University, Awatramani, [13]	RRID:MMRRC_069932-MU
	DAT-cre	Jackson Laboratory 006660	RRID:IMSR_JAX:006660
	Anxa1-icre	Transgenic and Targeted Mutagenesis Laboratory, Northwestern University Awatramani, [13]	RRID:MMRRC_069931-MU
	DATflpo	Transgenic and Targeted Mutagenesis Laboratory, Northwestern University Awatramani	
	Gt(ROSA)26Sorem1(EEF1A1-APX1*)Ykozo	MGI:7529474[46]	RRID:MGI:7529479
Eukaryotic cell lines	HEK-293T	ATCC	CRL-3216
Dataset	The Parkinson's disease kinase LRRK2 impairs release sites of vulnerable dopamine axons-APEX2 proteome	ProteomeXchange	PXD065003
	Mass spectrometry-synaptosome	ProteomeXchange	PXD065006
	The Parkinson's disease kinase LRRK2 impairs release sites of vulnerable dopamine axons	ProteomeXchange	PXD065125
	LRRK2 alters the composition and organization of active zone release sites in dopaminergic striatal synaptosomes	BioStudies	S-BIAD2074
	LRRK2 is expressed in dopamine neurons and is enriched in the vulnerable subpopulation	BioStudies	S-BIAD2070
	TH fibers and dopamine vesicle clustering alterations	BioStudies	S-BIAD2072
	LRRK2 disrupts the number and composition of active zone release sites in vulnerable dopamine axons	BioStudies	S-BIAD2071
	Source data	Zenodo	10.5281/zenodo.19930567
Software	Imaris 10.1	Bitplane Inc	RRID:SCR_007370
	GraphPad Prism 10	GraphPad Software Inc	RRID:SCR_002798
	ImageJ	doi:10.1038/nmeth.2089	RRID:SCR_003070
	iBright CL1500 Imaging System	Invitrogen	RRID:SCR_026565
	DIA-NN 1.9.2	https://github.com/vdemichev/DiaNN	RRID:SCR_022865
	CURTAIN	https://curtain.proteo.info	RRID:SCR_024465
	Limma	R software package	RRID:SCR_010943
	Jupyter Notebook	https://jupyter.org/	RRID:SCR_018315
	Axon Digidata 1550B	Molecular Devices	
	MATLAB	The MathWorks, Inc.	RRID:SCR_001622
	Doric Neuroscience Studio 5.4.1	Doric Lenses	RRID:SCR_018569
	Proteome Discoverer	Thermo Fisher Scientific	RRID:SCR_014477
	Spectronaut	Biognosys	
	Python	https://www.python.org/	RRID:SCR_008394
	Pandas	Python package	RRID:SCR_018214
	Matplotlib	Python 2D plotting library	RRID:SCR_008624
	seaborn	Python tool	RRID:SCR_018132

	NumPy	Python Package	RRID:SCR_008633
	Microsoft Excel	Microsoft	RRID:SCR_016137
	clusterProfiler	R package	RRID:SCR_016884
	org.Mm.eg.db	org.Mm.eg.db	RRID:SCR_023488
	readxl	R package	RRID:SCR_018083
	openxlsx	R package	RRID:SCR_019185
	dplyr	R package	RRID:SCR_016708
	ggplot2	R package	RRID:SCR_014601
	ComplexHeatmap	R package	RRID:SCR_017270
	NIS-Elements	Nikon Instruments Inc	RRID:SCR_014329
	LabVIEW	National Instruments	RRID:SCR_014325
Code	analysis code-phosphopeptidesGSMLi2vsGSvehpublication	Zenodo	10.5281/zenodo.19930567
	analysis code-volcanoPlots	Zenodo	10.5281/zenodo.19930567
	analysis code-WTvsLRRK2MLI2publication	Zenodo	10.5281/zenodo.19930567
	lrrk2_photometry_analysis	Zenodo/GitHub	10.5281/zenodo.20244434 https://github.com/DombeckLab/lrrk2_photometry_analysis
Protocol	Immunofluorescence and confocal imaging in brain sections and image analysis	protocols.io	dx.doi.org/10.17504/protocols.io.yxmvmm6q6v3p/v2
	Axonal proteome analysis and Western Blot via APEX2 labeling	protocols.io	dx.doi.org/10.17504/protocols.io.n2bvjb79bgk5/v2
	Liquid Chromatography-Mass Spectrometry (LC-MS) with striatal synaptosomes	protocols.io	dx.doi.org/10.17504/protocols.io.eq2lyqk6mvx9/v2
	SIM imaging and analysis for active zone release sites in vulnerable dopamine axons	protocols.io	dx.doi.org/10.17504/protocols.io.ewov1mpdpvr2/v2
	Western blot and Phostag gels	protocols.io	dx.doi.org/10.17504/protocols.io.j8nlkrxd5v5r/v2
	Striatal synaptosome preparation for imaging	protocols.io	dx.doi.org/10.17504/protocols.io.dm6gpqpo1lzp/v2
	Experiments in HEK-293 cells	protocols.io	dx.doi.org/10.17504/protocols.io.6qpvrqjm2lmk/v1
	Expression and Purification of FLAG-RAB3A Phosphorylated at pThr86	protocols.io	dx.doi.org/10.17504/protocols.io.dm6gpq4ejlzp/v1
	FLAG-tag mediated co-immunoprecipitation of phosphorylated Rab GTPases with cellular interacting proteins V.2	protocols.io	dx.doi.org/10.17504/protocols.io.5qpvowjn9l4o/v2
	Proteomics Sample Preparation for Affinity-Purification Mass Spectrometry	protocols.io	dx.doi.org/10.17504/protocols.io.8epv5owj6g1b/v1
	Surgical procedures for simultaneous optogenetic manipulation in the SNc and acute fiber photometry in the dorsal striatum	protocols.io	dx.doi.org/10.17504/protocols.io.e6nvwd6qdlmk/v1
