# Supplementary material for: Leucine-rich repeat kinase 2 impairs the release sites of Parkinson’s disease vulnerable dopamine axons
Source: Nat Commun. 2026 Jul 28;17:9165. doi: 10.1038/s41467-026-75194-3 (PMC13522591; doi:10.1038/s41467-026-75194-3)
Supplement: Supplementary file 2 [file 41467_2026_75194_MOESM2_ESM.pdf]

Description of Additional Supplementary Files

Supplementary Data 1: LC–MS/MS analysis of striatal synaptosomes isolated by subcellular fractionation from LRRK2^{G2019S} mice treated with MLi-2 or vehicle. Separate sheets contain inventories of phosphoproteins and phosphopeptides, as well as the input data used to generate the volcano plots.

Supplementary Data 2: Affinity mass spectrometry analysis defining the RAB3A and phosphorylated RAB3A (pRAB3A) interactomes. Pull-down samples contain proteins in the mouse brain associated with either RAB3A or pRAB3A. Separate sheets report individual sample protein abundances and differential-abundance analyses identifying proteins preferentially enriched in the RAB3A versus pRAB3A interactome.

Supplementary Data 3: APEX2 proximity-labeling DIA LC–MS/MS analysis of streptavidin pull-down samples from substantia nigra pars compacta (SNc) and striatum of Anxa1^{Cre} mice expressing LRRK2^{WT} or LRRK2^{G2019S}, treated with vehicle or MLi-2, along with Anxa1-negative LRRK2^{G2019S} control mice. Separate sheets contain protein, peptide, and phosphosite inventories; quantitative intensities; and results of group comparisons.

Supplementary Data 4: Gene Ontology analysis was performed on proteins containing at least one differentially regulated phosphopeptide in striatal synaptosomes from vehicle- and MLi2-treated LRRK2^{G2019S} mice. A full list of enriched Biological Process (BP), Cellular Component (CC), and Molecular Function (MF) pathways is provided in separate sheets.

Supplementary Data 5: Gene Ontology analysis of proteins differentially expressed in APEX2 streptavidin pulldown striatum samples from vehicle vs. MLi2-treated Anxa1iCre LRRK2^{G2019S} mice. Full lists of enriched Biological Process, Cellular Component, and Molecular Function pathways are provided in separate sheets.
