# Supplementary material for: Leucine-rich repeat kinase 2 impairs the release sites of Parkinson’s disease vulnerable dopamine axons
Source: Nat Commun. 2026 Jul 28;17:9165. doi: 10.1038/s41467-026-75194-3 (PMC13522591; doi:10.1038/s41467-026-75194-3)
Supplement: Supplementary file 3 [file 41467_2026_75194_MOESM8_ESM.pdf]

nature portfolio

Corresponding author(s): Loukia Parisiadou

Last updated by author(s): Sep 11, 2025

Reporting Summary

Nature Portfolio wishes to improve the reproducibility of the work that we publish. This form provides structure for consistency and transparency in reporting. For further information on Nature Portfolio policies, see our Editorial Policies and the Editorial Policy Checklist.

Statistics

For all statistical analyses, confirm that the following items are present in the figure legend, table legend, main text, or Methods section.

n/a	Confirmed	
☐	☒	The exact sample size (*n*) for each experimental group/condition, given as a discrete number and unit of measurement
☐	☒	A statement on whether measurements were taken from distinct samples or whether the same sample was measured repeatedly
☐	☒	The statistical test(s) used AND whether they are one- or two-sided<br>*Only common tests should be described solely by name; describe more complex techniques in the Methods section.*
☐	☒	A description of all covariates tested
☐	☒	A description of any assumptions or corrections, such as tests of normality and adjustment for multiple comparisons
☐	☒	A full description of the statistical parameters including central tendency (e.g. means) or other basic estimates (e.g. regression coefficient) AND variation (e.g. standard deviation) or associated estimates of uncertainty (e.g. confidence intervals)
☐	☒	For null hypothesis testing, the test statistic (e.g. *F*, *t*, *r*) with confidence intervals, effect sizes, degrees of freedom and *P* value noted<br>*Give P values as exact values whenever suitable.*
☒	☐	For Bayesian analysis, information on the choice of priors and Markov chain Monte Carlo settings
☒	☐	For hierarchical and complex designs, identification of the appropriate level for tests and full reporting of outcomes
☐	☒	Estimates of effect sizes (e.g. Cohen's *d*, Pearson's *r*), indicating how they were calculated

Our web collection on statistics for biologists contains articles on many of the points above.

Software and code

Policy information about availability of computer code

Data collection	Nikon Elements software (NIS-Elements) Nikon Inc. RRID:SCR_014329<br>iBright CL1500 Imaging System Invitrogen RRID:SCR_026565<br>Axon Digidata 1550B Molecular Devices<br>LabVIEW National Instruments RRID:SCR_014325<br>Doric Neuroscience Studio 5.4.1 Doric Lenses RRID:SCR_018569<br>Thermo Scientific Orbitrap Exploris 480 mass spectrometer (Thermo Scientific)
Data analysis	IImaris 10.1 Bitplane Inc RRID:SCR_007370<br>GraphPad Prism 10 GraphPad Software Inc RRID:SCR_002798<br>ImageJ doi:10.1038/nmeth.2089 RRID:SCR_003070<br>iBright Analysis Software Invitrogen RRID:SCR_017632<br>DIA-NN 1.9.2 https://github.com/vdemichev/DiaNN RRID:SCR_022865<br>CURTAIN https://curtain.proteo.info RRID:SCR_024465<br>Limma R software package RRID:SCR_010943<br>Jupyter Notebook https://jupyter.org/ RRID:SCR_018315<br>Axon Digidata 1550B Molecular Devices<br>MATLAB The MathWorks, Inc. RRID:SCR_001622<br>Doric Neuroscience Studio 5.4.1 Doric Lenses RRID:SCR_018569<br>LabVIEW National Instruments RRID:SCR_014325

Proteome Discoverer Thermo Fisher Scientific RRID:SCR_014477
Spectronaut Biognosys
Python https://www.python.org/ RRID:SCR_008394
Pandas Python package RRID:SCR_018214
Matplotlib Python 2D plotting library RRID:SCR_008624
seaborn Python tool RRID:SCR_018132
NumPy Python Package RRID:SCR_008633
Microsoft Excel Microsoft RRID:SCR_016137
clusterProfiler R package RRID:SCR_016884
org.Mm.eg.db org.Mm.eg.db RRID:SCR_023488
readxl R package RRID:SCR_018083
openxlsx R package RRID:SCR_019185
dplyr R package RRID:SCR_016708
ggplot2 R package RRID:SCR_014601
ComplexHeatmap R package RRID:SCR_017270
NIS-Elements Nikon Instruments Inc RRID:SCR_014329

Code availability
The codes generated in this study have been deposited on Zenodo, https://doi.org/10.5281/zenodo.19930567 ; https://doi.org/10.5281/zenodo.20244434, and GitHub, https://github.com/DombeckLab/lrrk2_photometry_analysis.
analysis code-phosphopeptidesGSMLi2vsGSvehpublication Zenodo https://doi.org/10.5281/zenodo.19930567
analysis code-volcanoPlots Zenodo https://doi.org/10.5281/zenodo.19930567
analysis code-WTvsLRRK2MLI2publication Zenodo https://doi.org/10.5281/zenodo.19930567
lrrk2_photometry_analysis Zenodo:10.5281/zenodo.20244434 GitHub: https://github.com/DombeckLab/lrrk2_photometry_analysis

For manuscripts utilizing custom algorithms or software that are central to the research but not yet described in published literature, software must be made available to editors and reviewers. We strongly encourage code deposition in a community repository (e.g. GitHub). See the Nature Portfolio guidelines for submitting code & software for further information.

Data

Policy information about availability of data

All manuscripts must include a data availability statement. This statement should provide the following information, where applicable:

- Accession codes, unique identifiers, or web links for publicly available datasets
- A description of any restrictions on data availability
- For clinical datasets or third party data, please ensure that the statement adheres to our policy

NEW DATASETS:
The mass spectrometry proteomics data generated in this study have been deposited in the ProteomeXchange Consortium via the PRIDE partner repository, with dataset identifiers PXD065003 (https://www.ebi.ac.uk/pride/archive/projects/PXD065003), PXD065006 (https://www.ebi.ac.uk/pride/archive/projects/PXD065006), and PXD0651255 (https://www.ebi.ac.uk/pride/archive/projects/PXD065125). The processed proteomics data generated in this study are provided in the Supplementary data 1-3, and Gene Ontology analysis results are provided in the Supplementary data 4-5. Imaging data with metadata files generated in this study have been deposited in the BioStudies/BioImage Archive, with data indentifiers S-BIAD2070 (https://www.ebi.ac.uk/biostudies/bioimages/studies/S-BIAD2070), S-BIAD2071 (https://www.ebi.ac.uk/biostudies/bioimages/studies/S-BIAD2071), S-BIAD2072 (https://www.ebi.ac.uk/biostudies/bioimages/studies/S-BIAD2072), and S-BIAD2074 (https://www.ebi.ac.uk/biostudies/bioimages/studies/S-BIAD2074). Raw tubular Data generated in this study from all experiments, and uncropped western blot images with metadata files in this study are available in source data file via the open-access option on the Zenodo repository (https://doi.org/10.5281/zenodo.19930567). Source data are provided with this paper. The protocols used in this study were uploaded to protocols.io; their DOIs are listed in the key resources table.

EXISTING DATASETS.
Kamath et al. snRNAseq data GEO: GSE178265
Saunders et al. RNAseq data: http://dropviz.org/

Research involving human participants, their data, or biological material

Policy information about studies with human participants or human data. See also policy information about sex, gender (identity/presentation), and sexual orientation and race, ethnicity and racism.

Reporting on sex and gender	N/A
Reporting on race, ethnicity, or other socially relevant groupings	N/A
Population characteristics	N/A

Recruitment	N/A
Ethics oversight	N/A

Note that full information on the approval of the study protocol must also be provided in the manuscript.

Field-specific reporting

Please select the one below that is the best fit for your research. If you are not sure, read the appropriate sections before making your selection.

[x] Life sciences [ ] Behavioural & social sciences [ ] Ecological, evolutionary & environmental sciences

For a reference copy of the document with all sections, see nature.com/documents/nr-reporting-summary-flat.pdf

Life sciences study design

All studies must disclose on these points even when the disclosure is negative.

Sample size	Sample size was determined using G power and prior experience.
Data exclusions	In the in vivo evoked dopamine release experiments: Post-hoc histological verification of virus expression and fiber placement were used as exclusion criteria, which led to the exclusion of one G2019S mouse due to the abnormality of GRAB-DA3m and ChRmine expression.
Replication	All experiments were performed at least three times.
Randomization	Littermates were randomly assigned to experimental procedures
Blinding	Researcher blinding was implemented across experiments.

Reporting for specific materials, systems and methods

We require information from authors about some types of materials, experimental systems and methods used in many studies. Here, indicate whether each material, system or method listed is relevant to your study. If you are not sure if a list item applies to your research, read the appropriate section before selecting a response.

Materials & experimental systems

n/a	Involved in the study	
[ ]	[x]	Antibodies
[ ]	[x]	Eukaryotic cell lines
[x]	[ ]	Palaeontology and archaeology
[ ]	[x]	Animals and other organisms
[x]	[ ]	Clinical data
[x]	[ ]	Dual use research of concern
[x]	[ ]	Plants

Methods

n/a	Involved in the study	
[x]	[ ]	ChIP-seq
[x]	[ ]	Flow cytometry
[x]	[ ]	MRI-based neuroimaging

Antibodies

Antibodies used

phospho-RAB12 (S106) Abcam, ab256487 1:1000
RAB12 Proteintech, 18843-1-AP 1:1000
β-Actin Sigma-Aldrich, A5441 1:3000
LRRK2/Dardarin cloneN241A/34 NeuroMab, 75-253 1:200
LRRK2/Dardarin cloneN137/6 NeuroMab, 75-188 1:200
LRRK2 (phospho S935) Abcam, ab1334501 :1000
LRRK2 (phospho S1292) Abcam, ab203181 1:500
Streptavidin-HRP Abcam, ab7403 1:1000
RAB3A Sigma-Aldrich, WH0005864M1 1:1000
RAB3C Proteintech, 10788-1-AP 1:1000
Synaptophysin Cell signaling, 4329 1:1000
Bassoon (SAP7F407) Enzo, ADI-VAM-PS003 1:300
Tyrosine hydroxylase SYSY, 213-104 1:1000
Tyrosine hydroxylase Millipore, MAB318 1:1000
ALDH1A1 Abcam, ab52492 1:500
ALDH1A1 Proteintech, 60171-1-Ig 1:500
anti-GFP Invitrogen, A10262 1:500
anti-GFP Abcam, ab6556 1:500

anti-mCherry Invitrogen, M11217 1:500
VAMP2 R&D systems, AF5136 1:1000
SNAP25 Proteintech, 14903-1-AP 1:1000
Dopamine Transporter Sigma-Aldrich, MAB369 1:1000
RIM1 Proteintech, 24576-1-AP 1:1000
GDI1 Sigma-Aldrich, WH0002664M8 1:1000
GDI2 Novus, NBP3-03650 1:1000
VMAT2 Immunostar, 20042 1:500
GAPDH (6C5) Santa Cruz, Sc-32233 1:500
anti-Flag Sigma, F1804 1:1000
anti--HA Roche, 11867423001 1:500
anti-c-myc Sigma, M4439 1:2000

Validation

The majority of antibodies used in this study are validated either through KD or KO approaches. Number of publications are noted and two of them are given as example.

phospho-RAB12 (S106), RRID:AB_2884880: 27 citations. Validated with KO PMID: 37874635. WB; tested species: mouse (PMID: 40038582,PMID: 34340748)
RAB12, RRID:AB_10603469: 24 citations. KD/KO Validated (PMID: 26740112, PMID: 37874635) WB; tested species: mouse (PMID: 37874635, PMID: 39514635)
β-Actin ,RRID:AB_476744: 12658 citations. KO Validated (PMID: 29170425) WB; tested species: mouse (PMID: 40394007,PMID: 40394007)
LRRK2/Dardarin cloneN241A/34, RRID:AB_10675136: 127 citations. Validated with KO in manufacturer's webpage and in Supplementary Figure 1. ICC-IF, WB; tested species: mouse (PMID: 33135724, PMID: 37289222)
LRRK2/Dardarin cloneN137/6, RRID:AB_2234791: 23 citations. Validated with KO in manufacturer's webpage. WB; tested species: mouse (PMID: 28103901, PMID: 27273569)
LRRK2 (phospho S935), RRID:AB_2732035: 119 citations. This antibody was developed with the support of the Michael J. Fox Foundation and in partnership with Dr. Alessi from the MRC Phosphorylation Unit at the University of Dundee. It has been validated with Lrrk2 knockout (KO) tissue in both the literature and in Supplementary Figure 1 of this study. Additionally, it has been further validated by several research groups (PMID: 33741046) using various specific LRRK2 kinase inhibitors, including those in our own publications (PMID: 38895420). WB; tested species: mouse (PMID: 24464040, PMID: 26365310)
LRRK2 (phospho S1292), RRID:AB_2921223: 70 citations. KO validated. Supplementary Figure 1. WB; tested species: mouse (PMID: 28292328, PMID: 29707617)
RAB3A, RRID:AB_1843230: 3 citations. Validated in Supplementary Figure 2J of this study by using overexpressed plasmids that have been sequenced and confirmed. WB; tested species: mouse (PMID: 30483077)
RAB3C, RRID:AB_2177217: 4 citations. Validated in our Supplementary Figure 2J of this study by using overexpressed plasmids that have been sequenced and confirmed. WB; tested species: mouse (PMID: 19717423)
Synaptophysin, RRID:AB_1904154: 50 citations. IF, WB; tested species: mouse (PMID: 30728170, PMID: 35799541)
Bassoon (SAP7F407), RRID:AB_10618753: 140 citations. IF; tested species: mouse (PMID: 29398114, PMID: 34767769)
Tyrosine hydroxylase (SYSY), RRID:AB_2619897: 17 citations. IHC, WB; tested species: mouse (PMID: 39738072, PMID: 34767769)
Tyrosine hydroxylase (Millipore), RRID:AB_2313764: 1181 citations. IHC, IF, WB; tested species: mouse (PMID: 29398114, PMID: 34767769)
ALDH1A1 (ABcame), RRID:AB_867566: 214 citations. Manufacturer's website"The specificity of Anti-ALDH1A1 antibody [EP1933Y] - C-terminal (ab52492) has been confirmed by Western blot testing in ALDH1A1 Knockout A549 cells." IF, WB; tested species: mouse (PMID: 30504836, PMID: 38355624)
ALDH1A1 (Proteintech), RRID:AB_10693634:18 citations. KD validated (PMID: 37867947). IF; tested species: mouse (PMID: 38470102)
anti-GFP (Invitrogen), RRID:AB_2534023: 428 citations. IF; tested species: mouse (PMID: 36693373,PMID: 35022617) Advanced verification through relative expression to ensure that the antibody binds to the antigen stated. From manufacturer's webpage: "Antibody specificity was demonstrated by the detection of different targets fused to a GFP tag in transiently transfected lysates. Relative detection of GFP tag was observed across different proteins fused with GFP..."
anti-GFP (Abcam), RRID:AB_305564: 1598 citations. IF; tested species: mouse (PMID: 28220790, PMID: 31315053)
anti-mCherry, RRID:AB_2536611: 424 citations. IF; tested species: mouse (PMID: 33247179, PMID: 32697988)
VAMP2, RRID:AB_10571702:1 citation. IHC; tested species: Rat (Manufacturer's website) WB; tested species: Homo sapiens (Human) (PMID: 36316316)
SNAP25, RRID:AB_2192051: 24 citations. KD validated (PMID: 35752613) WB; tested species: mouse (PMID: 34551807, PMID: 39277023)
Dopamine Transporter (DAT), RRID:AB_2190413: 383 citations. WB; tested species: mouse (PMID: 37433762, PMID: 33414501)
RIM1, RRID:AB_2879618: 6 citations. KD/KO Validated (PMID: 29033205) WB; tested species: mouse (PMID: 38218585,PMID: 29033205)
GDI1, RRID:AB_1841744: WB; tested species: Homo sapiens (Human) (manufacturer's webpage)
GDI2, RRID:AB_3532284: Validated with KO in manufacturer's webpage. WB; tested species: mouse (manufacturer's webpage).
VMAT2, RRID:AB_10013884: 9 citations. WB; tested species: Rat (manufacturer's webpage) IF, IHC; tested species: mouse (PMID: 25904808; PMID: 29398114). From manufacturer's webpage: "Specificity of the antiserum was demonstrated by soluble pre-adsorption and Western blot. Tissue staining is completely eliminated by pretreatment of the diluted antibody with an excess of rat VMAT2 peptide residues 496-515."
GAPDH (6C5), RRID:AB_627679: 4409 citations. WB; tested species: mouse (PMID: 39613744, PMID: 40562771)
anti-Flag, RRID:AB_262044: 10932 citations. WB; tested species: mouse (PMID: 40419468, PMID: 40419509) WB; tested species: Homo sapiens (Human) (PMID: 40360474)

anti--HA, RRID:AB_390918: 342 citations. WB; tested species: Homo sapiens (Human) (PMID: 39032489)
anti-c-myc, RRID:AB_439694. 1795 citations. WB; tested species: Homo sapiens (Human) (PMID: 37952051,PMID: 31434890) WB; tested species: mouse (PMID: 31434890)
Streptavidin-HRP, 178 citations. WB; tested species: mouse (PMID: 25306109, PMID: 28437250)

Eukaryotic cell lines

Policy information about cell lines and Sex and Gender in Research

Cell line source(s)	HEK-293T (ATCC, CRL-3216)
Authentication	Short tandem repeat profiling, daily inspection of morphology
Mycoplasma contamination	Cell line used in this study was confirmed free of mycoplasma contamination.
Commonly misidentified lines (See ICLAC register)	HeLa

Animals and other research organisms

Policy information about studies involving animals; ARRIVE guidelines recommended for reporting animal research, and Sex and Gender in Research

Laboratory animals

LRRK2 G2019S Jackson Laboratory 030961 RRID:IMSR_JAX:030961
C57BL/6 Jackson Laboratory 000664 RRID:IMSR_JAX:000664
LRRK2 KO Jackson Laboratory 016121 RRID:IMSR_JAX:016121
Aldh1a1-cre Transgenic and Targeted Mutagenesis Laboratory, Northwestern University, Awatramani, PMID: 37537242
DAT-cre Jackson Laboratory 006660 RRID:IMSR_JAX:006660
Anxa1-icre Transgenic and Targeted Mutagenesis Laboratory, Northwestern University Awatramani, PMID: 37537242
DATflpo Transgenic and Targeted Mutagenesis Laboratory, Northwestern University Awatramani
Gt(ROSA)26Sorem1(EEF1A1-APX1*)Ykozo MGI:752947444 RRID:MGI:7529479

Adult mice, aged 3 to 7 months, were used in all experiments.

Wild animals

Provide details on animals observed in or captured in the field; report species and age where possible. Describe how animals were caught and transported and what happened to captive animals after the study (if killed, explain why and describe method; if released, say where and when) OR state that the study did not involve wild animals.

Reporting on sex	Both male and female mice were used in all experiments. No sex-based analysis was performed.
Field-collected samples	The mice were group-housed on a standard 12-hour light/dark cycle and provided with standard feed.
Ethics oversight	All experiments complied with the guidelines set by the National Institutes of Health and were reviewed by the Northwestern Animal Care and Use Committee or University of Dundee ethical committee.

Note that full information on the approval of the study protocol must also be provided in the manuscript.

Plants

Seed stocks	N/A
Novel plant genotypes	N/A
Authentication	N/A
