# Supplementary material for: Leucine-rich repeat kinase 2 impairs the release sites of Parkinson’s disease vulnerable dopamine axons
Source: Nat Commun. 2026 Jul 28;17:9165. doi: 10.1038/s41467-026-75194-3 (PMC13522591; doi:10.1038/s41467-026-75194-3)
Supplement: Supplementary file 4 [file 41467_2026_75194_MOESM9_ESM.pdf]

nature portfolio

Peer Review File

Leucine-rich repeat kinase 2 impairs the release sites of Parkinson's disease vulnerable dopamine axons

Corresponding Author: Professor Loukia Parisiadou

This manuscript has been previously reviewed at another journal. This document only contains information relating to versions considered at Nature Communications.

This file contains all reviewer reports in order by version, followed by all author rebuttals in order by version.

Version 0:

Reviewer comments:

Reviewer #1

(Remarks to the Author)
The revised manuscript has been significantly improved and is appropriate for publication in Nat Comm. The most significant contribution of this work is to provide solid, conclusive data demonstrating LRRK2 expression in nigral DA neurons, particularly in the most vulnerable subpopulation, which has puzzled the field for some time. This paper will be of great interest to researchers in the PD field and those who study LRRK biology.

Reviewer #3

(Remarks to the Author)
This manuscript investigates how pathogenic LRRK2 drives early synaptic dysfunction in Parkinson's disease by focusing on vulnerable dopamine neuron subpopulations. Using a combination of intersectional genetic models, proximity labeling proteomics, phosphoproteomics, imaging, and in vivo dopamine measurements, the authors show that LRRK2 is enriched in vulnerable dopamine neurons, increases phosphorylation of RAB3 proteins, disrupts their interaction with RIM1/2, alters active zone organization, and ultimately reduces dopamine release in vivo. Overall, the study is well designed with very interesting findings in a physiologically relevant mouse model. I have a few comments about the proteomics experiments in this paper:

1. To label DA neuron-specific proteome, the authors expressed APEX2 in SNs. Biotinylation signals and correct location was validated by imaging and western. APEX2 proteomics was conducted and compared with published dopamine neuron proteomics results in Fig S1F. Did the author detect LRRK2 protein directly from the proteomics results? I found it odd to conduct APEX2 proteomics but then report the detection of LRRK2 protein by a faint signal in western blot instead of using LC-MS data which is more sensitive and unbiased.

2. A follow-up on the above comment, how many total proteins were quantified in the APEX data and was there any control group for APEX proteomics to use volcano plot with statistical information rather than a ranking plot?

3. The authors reported decreased phosphorylation of RAB3 T86 in line 188. However, in Figure 2B, RAB3 T86 appears to be increased on the right side of the volcano plot? Also can the authors label other significantly changed peptides in the volcano plots, at least for RAB3A and RAB3C which were claimed to be the key findings with highest expression levels?

4. To evaluate the quality of the proteomics results, the authors need to provide complete proteomics results (APEX, phosphoproteomics, and IP-MS) in excel as supplementary tables to include at least the protein information, # unique peptides, abundance of each sample, and statistical results.

5. It is unclear how many biological replicates were conducted for omics experiments, and whether and how samples were pooled together. Can the authors provide the number of biological replicates in all relevant figure legends?

Version 1:

Reviewer comments:

Reviewer #3

(Remarks to the Author)
The authors have addressed all my comments.

Open Access This Peer Review File is licensed under a Creative Commons Attribution 4.0 International License, which permits use, sharing, adaptation, distribution and reproduction in any medium or format, as long as you give appropriate credit to the original author(s) and the source, provide a link to the Creative Commons license, and indicate if changes were made.
In cases where reviewers are anonymous, credit should be given to 'Anonymous Referee' and the source.
The images or other third party material in this Peer Review File are included in the article's Creative Commons license, unless indicated otherwise in a credit line to the material. If material is not included in the article's Creative Commons license and your intended use is not permitted by statutory regulation or exceeds the permitted use, you will need to obtain permission directly from the copyright holder.
To view a copy of this license, visit https://creativecommons.org/licenses/by/4.0/

Rebuttal Letter

We sincerely thank the Reviewers for their thoughtful and constructive feedback on our manuscript, "Leucine-rich repeat kinase 2 impairs the release sites of Parkinson's disease vulnerable dopamine axons." We are particularly encouraged by their agreement that our manuscript has significantly improved and is of strong interest to the Parkinson's disease (PD) field. We have addressed all remaining comments, with changes marked in blue in the revised manuscript. Below, we provide a point-by-point response to the Reviewer's comments.

Reviewer #2 (Remarks to the Author):

The revised manuscript has been significantly improved and is appropriate for publication in Nat Comm. The most significant contribution of this work is to provide solid, conclusive data demonstrating LRRK2 expression in nigral DA neurons, particularly in the most vulnerable subpopulation, which has puzzled the field for some time. This paper will be of great interest to researchers in the PD field and those who study LRRK biology.

We sincerely thank the Reviewer for the very positive assessment of our revised manuscript, for their supportive comments, and for viewing the work as appropriate for publication in *Nature Communications*.

Reviewer #3 (Remarks to the Author):

This manuscript investigates how pathogenic LRRK2 drives early synaptic dysfunction in Parkinson's disease by focusing on vulnerable dopamine neuron subpopulations. Using a combination of intersectional genetic models, proximity labeling proteomics, phosphoproteomics, imaging, and in vivo dopamine measurements, the authors show that LRRK2 is enriched in vulnerable dopamine neurons, increases phosphorylation of RAB3 proteins, disrupts their interaction with RIM1/2, alters active zone organization, and ultimately reduces dopamine release in vivo. Overall, the study is well designed with very interesting findings in a physiologically relevant mouse model. I have a few comments about the proteomics experiments in this paper:

We appreciate the Reviewer's evaluation of the proteomics aspects of this study. The constructive comments from this Reviewer have helped us improve the clarity, rigor, and reporting of the omics data.

1. To label DA neuron-specific proteome, the authors expressed APEX2 in SNs. Biotinylation signals and correct location was validated by imaging and western. APEX2 proteomics was conducted and compared with published dopamine neuron proteomics results in Fig S1F. Did the author detect LRRK2 protein directly from the proteomics results? I found it odd to conduct APEX2 proteomics but then report the detection of LRRK2 protein by a faint signal in western blot instead of using LC-MS data which is more sensitive and unbiased.

We thank the Reviewer for this important comment. The APEX2 experiment was designed as a discovery proteomics approach to define the dopamine neuron-enriched proteome in a cell-type-specific manner. Although liquid chromatography–mass spectrometry (LC-MS) is a powerful and unbiased platform, protein detection in discovery datasets can still be influenced by protein abundance and peptide chemo-physical properties, including their length, ionization efficiency, and MS detectability. These limitations are particularly relevant for large, low-abundance proteins such as LRRK2. In addition, extensive post-translational modification, including phosphorylation, may further reduce detectability (PMIDs: 19607789;35172570).

In the current study, we applied High-Field Asymmetric Waveform Ion Mobility Spectrometry–Data Independent Acquisition (FAIMS-DIA) with high-resolution mass spectrometry. FAIMS may improve signal-to-noise ratio and dynamic range, but may reduce global sequence coverage. As previously reported (PMID: 39820751), voltage-dependent incomplete peptide sampling can lead to the loss of abundant precursors and reduced peptide and sequence coverage. In addition, APEX2-induced biotinylation commonly occurs on lysine residues, which are also trypsin cleavage sites, potentially interfering with tryptic digestion and peptide generation.

Consistent with these limitations, LRRK2 was detected only at low levels and inconsistently across the APEX discovery datasets. We therefore deemed the LC-MS signal insufficiently robust to support conclusions about LRRK2 enrichment from the proteomics dataset alone. Instead, we used targeted approaches, including western blotting of APEX2-enriched samples with a specific LRRK2 antibody and independent immunofluorescence analyses, with LRRK2-knockout tissue as a stringent negative control. We have now clarified this rationale in the revised manuscript (**lines 394-398**).

2. A follow-up on the above comment, how many total proteins were quantified in the APEX data and was there any control group for APEX proteomics to use volcano plot with statistical information rather than a ranking plot?

We thank the Reviewer for this comment. Our APEX2 proteomics workflow included $APEX2^{-}$ controls to define proteins recovered independently of APEX2-mediated proximity labeling, including endogenously biotinylated proteins or nonspecifically retained proteins. Proteins not significantly enriched in $APEX2^{+}$ versus $APEX2^{-}$ samples were therefore filtered out from both the SNc and striatal datasets, as described in the Materials and Methods (lines 861–862) and Supplementary Figure 6 (lines 2162–2165). After this control-based filtering step, the final APEX-enriched dataset contained 63,495 peptides mapping to 5,567 proteins (**line** 2164).

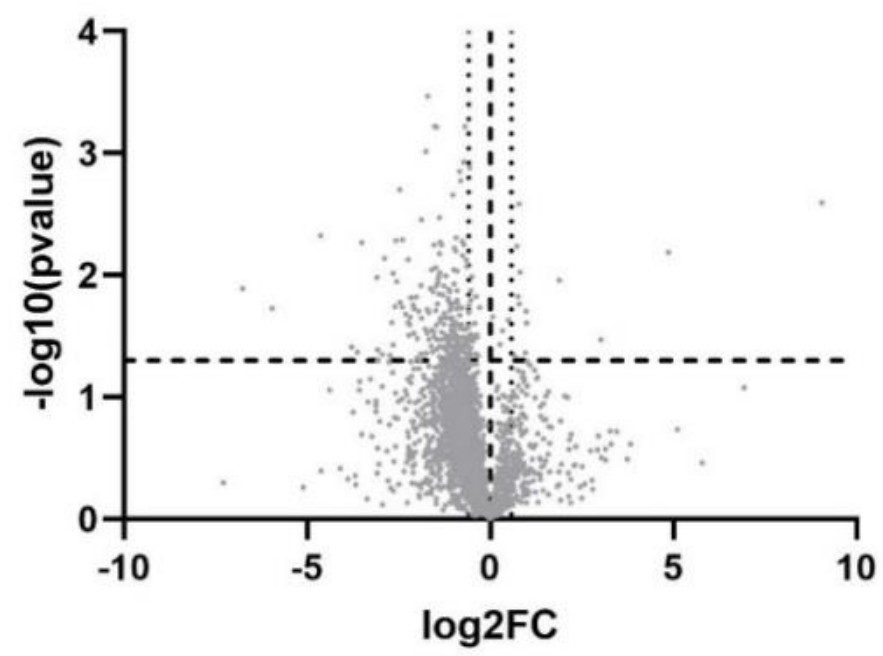

Rebuttal Figure 1: Volcano plot comparing the significantly altered total proteins from streptavidin pulldowns from the striatum ($|Log2FC| > 0.58$ and unadjusted p-value ≤ 0.05 by multiple unpaired t-tests) between vehicle and MLi-2-treated striatal $Anx1i^{cre}LRRK2^{G2019S}$ mouse injected with APEX2 AAV. Each sample is the eluted proteins from one mouse. vehicle n=7, MLi2 n=5. Significantly altered proteins were used for the GO enrichment analysis shown in Supplementary Figure 6E.

Importantly, the APEX-enriched proteomes were then subjected to formal statistical analyses across genotype and treatment comparisons, including $LRRK2^{WT}$ versus $LRRK2^{G2019S}$ and $LRRK2^{G2019S}$ + MLi-2 versus vehicle. Significantly altered proteins were defined using thresholds of $|log2FC| > 0.58$ and unadjusted $p \leq 0.05$. For example, Supplementary Figure 6E presents downstream Gene Ontology (GO) enrichment analysis of the treatment-specific, significantly altered proteins in $LRRK2^{G2019S}$ + MLi-2 compared with vehicle control. For the Reviewer's reference, we now provide the corresponding volcano plot as Rebuttal Figure 1. A similar statistical comparison was performed for $LRRK2^{WT}$ versus $LRRK2^{G2019S}$, and the complete list of significantly enriched proteins and peptides across all APEX proteomics comparisons is available in the PRIDE repository PXD065003 and in Supplementary File 3.

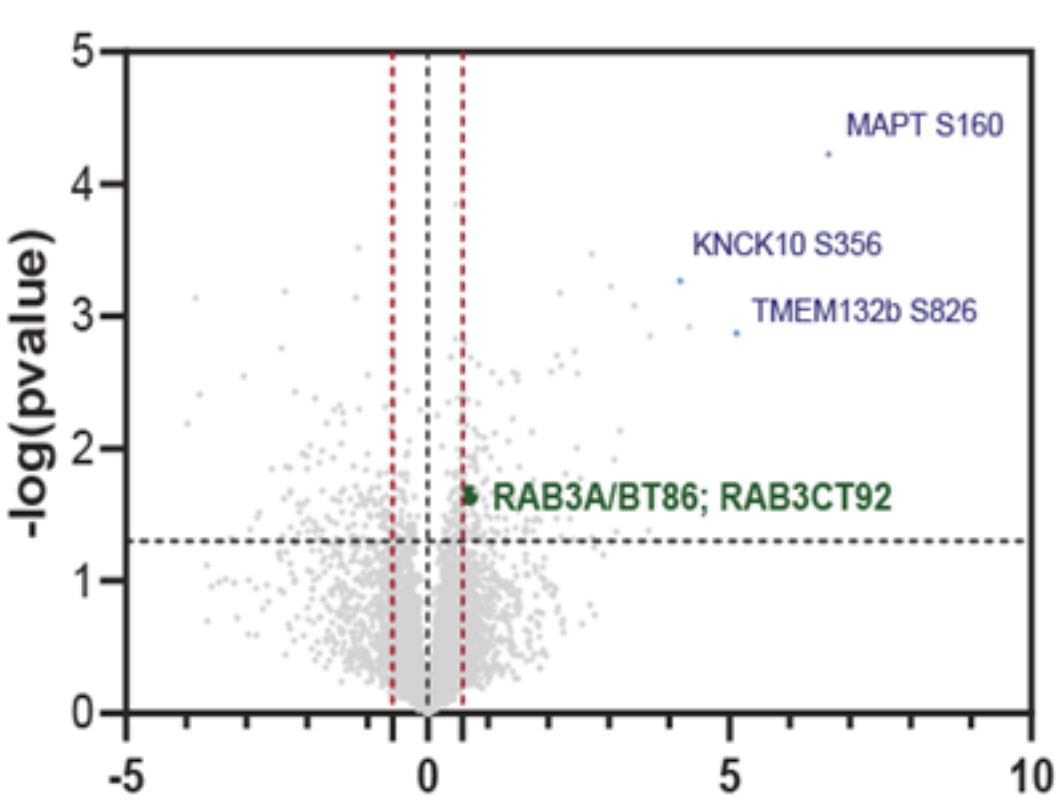

Rebuttal Figure 2: Volcano plot showing phosphopeptides altered between vehicle- and MLi-2-treated $LRRK2^{G2019S}$ striatal synaptosomes. Log2FC is plotted as vehicle relative to MLi-2, such that phosphopeptides on the right are higher in vehicle-treated samples. Significance was defined by multiple unpaired t-tests with $p \leq 0.05$ and $|log2FC| > 0.58$. The shared RAB3 phosphopeptide mapping to RAB3A/B T86 and RAB3C T92 is highlighted in green. Additional labeled significantly altered phosphopeptides are shown in blue. Each sample consisted of striatal synaptosomes pooled from 3 mice; n = 3

In contrast, the ranking plot shown in panel C of Supplementary Figure 6 was used only for proof-of-principle analysis, showing the enrichment of dopamine neuron markers across the overall dataset. We have now revised the text and figure legends to clarify these distinctions in the revised manuscript (**lines** 2159 and 2162-65).

3. The authors reported decreased phosphorylation of RAB3 T86 in line 188. However, in Figure 2B, RAB3 T86 appears to be increased on the right side of the volcano plot? Also can the authors label other significantly changed peptides in the volcano plots, at least for RAB3A and RAB3C which were claimed to be the key findings with highest expression levels?

We thank the Reviewer for pointing this out. The apparent discrepancy reflects the direction of the comparison shown in Figure 2B. In this volcano plot, log2FC is shown for $LRRK2^{G2019S}$ vehicle relative to $LRRK2^{G2019S}$ + MLi-2. Thus, phosphopeptides on the right side of the plot are increased in vehicle-treated $LRRK2^{G2019S}$ samples and decreased following MLi-2 treatment. Accordingly, the rightward position of the RAB3 phosphopeptide is consistent with our conclusion that LRRK2 kinase inhibition decreases RAB3 phosphorylation. We have revised the Figure 2 legend to make the direction of the comparison explicit (**lines** 1647-50). We also clarified that the labeled RAB3 phosphopeptide maps to a

conserved/shared sequence corresponding to RAB3A/B T86 and the homologous RAB3C T92 site, and we have annotated it accordingly in the volcano plot in Figure 2B (**lines** 1650-52).

For the Reviewer's reference, we labeled some highly altered phosphopeptides in the Rebuttal Figure 2. In the main manuscript Figure 2, we focused on annotating the RAB3 phosphopeptide central to the study's mechanism. At the same time, we provide the phosphopeptide list (input) and statistical data used for volcano plots from this comparison in Supplementary File 1 and in the PRIDE dataset PXD065006. Consistent with these phosphoproteomics results, the Phos-tag analyses in Figure 2D–F provide targeted validation in SNc tissue and support a kinase-dependent increase in RAB proteins phosphorylation.

4. To evaluate the quality of the proteomics results, the authors need to provide complete proteomics results (APEX, phosphoproteomics, and IP-MS) in excel as supplementary tables to include at least the protein information, # unique peptides, abundance of each sample, and statistical results.

We fully agree that complete tabulated proteomics results are important for evaluating data quality and transparency. Accordingly, we have provided the complete APEX2, phosphoproteomics, and IP-MS datasets as Excel/CSV supplementary files, including protein identifiers, numbers of unique peptides, sample-level abundance values, and statistical results, as new Supplementary Files 1–3.

The mass spectrometry proteomics data were also deposited to the ProteomeXchange Consortium via the PRIDE partner repository under dataset identifiers PXD065003, PXD065006, and PXD065125 at the initial submission. These datasets include relevant protein information, unique peptide counts, abundance values for each sample, and associated statistical analyses, and will be made publicly available upon publication.

For review purposes, access credentials are provided below:
The mass spectrometry proteomics data have also been deposited to the ProteomeXchange Consortium via the PRIDE partner repository under dataset identifiers PXD065003, PXD065006, and PXD065125 since the initial submission. These datasets include the relevant protein information, unique peptide counts, abundance values for each sample, and associated statistical analyses and will be made publicly available upon publication.

For review purposes, access credentials are provided below:
- **PXD065003** Username: reviewer_pxd065003@ebi.ac.uk; Password: oLJFUqE39Luk
- **PXD065006** Username: reviewer_pxd065006@ebi.ac.uk; Password: s1xeUI0TBuyU
- **PXD065125** Username: reviewer_pxd065125@ebi.ac.uk; Password: 2oDNX43ZQGqs

In addition, the complete GO analysis results have been uploaded to Zenodo and are included as Supplementary File 4 for the synaptosome experiment shown in Figure 2 and Supplementary File 5 for the APEX2 experiment shown in Supplementary Figure 6. Finally, as part of the original submission, we deposited other primary data files to support transparency and reproducibility. Image raw files with metadata were deposited in the BioImage Archive under identifiers S-BIAD2070, S-BIAD2071, S-BIAD2072, and S-BIAD2074, and full western blot images with metadata were deposited in Zenodo (https://doi.org/10.5281/zenodo.19930567). These resources are described in the Data Availability section.

5. It is unclear how many biological replicates were conducted for omics experiments, and whether and how samples were pooled together. Can the authors provide the number of biological replicates in all relevant legends?

We agree that this information should be clearly and easily located. We have therefore added the number of biological replicates and pooling details to all relevant figure legends and manuscript sections, including the Materials and Methods section, **lines** 759–760 and 811–812; Figure 1 legend, **line** 1631; Figure 2 legend, **lines** 1643–1645; Figure S1 legend, **lines** 1870, 1872, and 1876–1877; Figure S2 legend, **lines** 1960–1961; and Figure S6 legend, **lines** 2159, 2167–2168, and 2170–2171. All sample IDs and experimental group details are also included in the PRIDE repository.
